# Organometallic complexes as donors and non-fullerene acceptors for organic solar cells: DFT-guided design, structure–property relationships, and future directions

**DOI:** 10.1039/d6ra07089b

**Published:** 2026-10-27

**Authors:** Muhammad Usman Khan

**Affiliations:** a Department of Chemistry, University of Okara Okara-56300 Pakistan usman.chemistry@gmail.com usmankhan@uo.edu.pk

## Abstract

Computational screening has become indispensable to organic solar cell (OSC) design, allowing the electronic, optical, and charge-transport properties of a candidate to be predicted before synthesis. Organometallic complexes (OMCs) are especially well suited to this approach: their metal centers offer design handles-tunable frontier-orbital energies, strong spin–orbit coupling (SOC), and metal-to-ligand charge-transfer (MLCT) transitions that purely organic semiconductors lack. Long confined to donor and interlayer roles, OMCs have recently emerged as credible non-fullerene acceptors (NFAs) once functionalized with electron-withdrawing groups, yet their donor and acceptor chemistries have been reviewed in isolation rather than within a common density functional theory (DFT) framework. This review examines how DFT and its time-dependent extension (TD-DFT) guide the design of OMCs that function as both donors and NFAs. We assess the prevailing computational protocols-hybrid and range-separated functionals (B3LYP, CAM-B3LYP, ωB97XD, M06-2X), effective core potentials (LANL2DZ), and continuum solvation models (PCM, SMD) and the descriptors that link molecular structure to device performance: frontier molecular orbital (FMO) energies, reorganization and exciton-binding energies, and SOC matrix elements. The literature is organized by metal center-ferrocene (Fe), iridium (Ir), platinum (Pt), ruthenium (Ru), osmium (Os), and metalloporphyrin/phthalocyanine systems. Across the surveyed systems, DFT-computed band gaps reproduce experimental trends well, and an optimal acceptor LUMO window of −3.5 to −3.9 eV emerges. Within this window, we rationalize the record power conversion efficiencies of 19.24% (ternary Pt-based) and 15.25% (ternary Cu-porphyrin) devices. We distill these findings into transferable, metal-specific design rules alongside a candid assessment of method reliability. We close by charting the integration of machine learning with high-throughput DFT toward OMC-NFAs that exceed 20% efficiency with improved operational stability.

## Abstract 2

This review explores DFT/TD-DFT-guided design of organometallic donors and non-fullerene acceptors for organic solar cells, focusing on molecular design, charge-transfer mechanisms, and strategies for efficient, stable photovoltaic devices.

## Graphical abstract

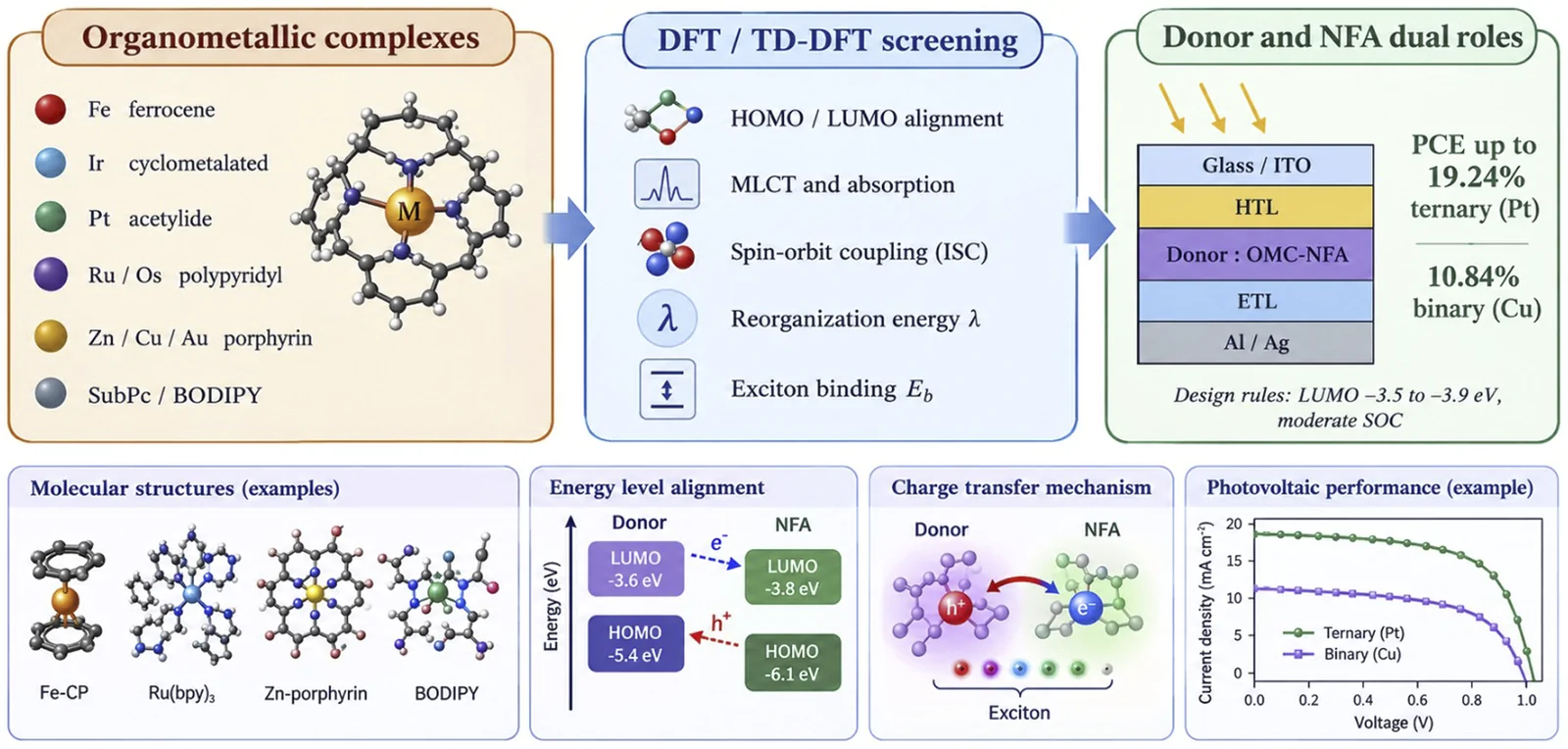

## 1 Introduction

The escalating global demand for clean, renewable energy has catalyzed intensive research into photovoltaic technologies that can complement or eventually replace silicon-based solar cells. Among emerging alternatives, organic solar cells (OSCs) have attracted considerable attention owing to their mechanical flexibility, lightweight nature, solution processability, semitransparency, and potential for low-cost roll-to-roll manufacturing. Over the past decade, the power conversion efficiencies (PCEs) of OSCs have surged from below 10% to beyond 20% in single-junction devices, driven primarily by the advent of non-fullerene acceptors (NFAs) such as the Y-series (Y6 and its derivatives).^1,2^ Despite these advances, further improvements in PCE, operational stability, and material diversity remain essential for the commercialization of OSC technology.

The working principle of OSCs is based on the photovoltaic effect in organic semiconductor thin films, where light absorption generates bound electron–hole pairs (excitons) that must be dissociated at a donor–acceptor (D/A) heterojunction to produce free charge carriers. The efficiency of this process depends critically on the optoelectronic properties of the active layer materials, including their absorption spectra, frontier molecular orbital (FMO) energy levels, charge carrier mobilities, exciton diffusion lengths, and the nanoscale morphology of the D/A blend.^3,4^ The design of new donor and acceptor materials with optimized properties remains the primary strategy for advancing OSC performance, and computational methods-particularly density functional theory have become essential tools for guiding this design process by enabling rapid virtual screening and mechanistic understanding of structure–property relationships before costly experimental synthesis and device fabrication.^5^

From this working principle, four fundamental requirements emerge that govern material design and that any predictive calculation must capture. (i) Light absorption: the optical gaps of the donor and acceptor should be complementary so that the blend harvests photons across the solar spectrum, with high oscillator strengths ensuring strong absorption in thin films. (ii) Energy-level alignment: the donor and acceptor frontier levels must be offset enough to drive interfacial electron and hole transfer, yet the |HOMO(donor)|–|LUMO(acceptor)| difference should remain as large as possible because it sets the attainable open-circuit voltage, so minimizing any excess offset reduces voltage losses. (iii) Charge-transfer character: photoexcitation initially produces a strongly bound Frenkel exciton whose binding energy (typically 0.2–0.4 eV in organic semiconductors) must be overcome at the D/A interface *via* interfacial charge-transfer states; the degree of intramolecular charge-transfer character, which is directly accessible from TD-DFT, correlates with smaller exciton binding energies and easier dissociation. (iv) Excited-state dynamics and charge separation: the exciton must diffuse to an interface within its lifetime, dissociate into a charge-transfer state, and then separate into free carriers before geminate recombination. In organometallic complexes this picture acquires an additional dimension, because spin–orbit coupling at the metal center opens intersystem crossing to long-lived triplet excitons that can extend the effective exciton diffusion length, but can also introduce triplet-mediated recombination channels. These interlinked processes and the corresponding energy-level requirements are summarized schematically in Fig. 1b.

**Fig. 1:**
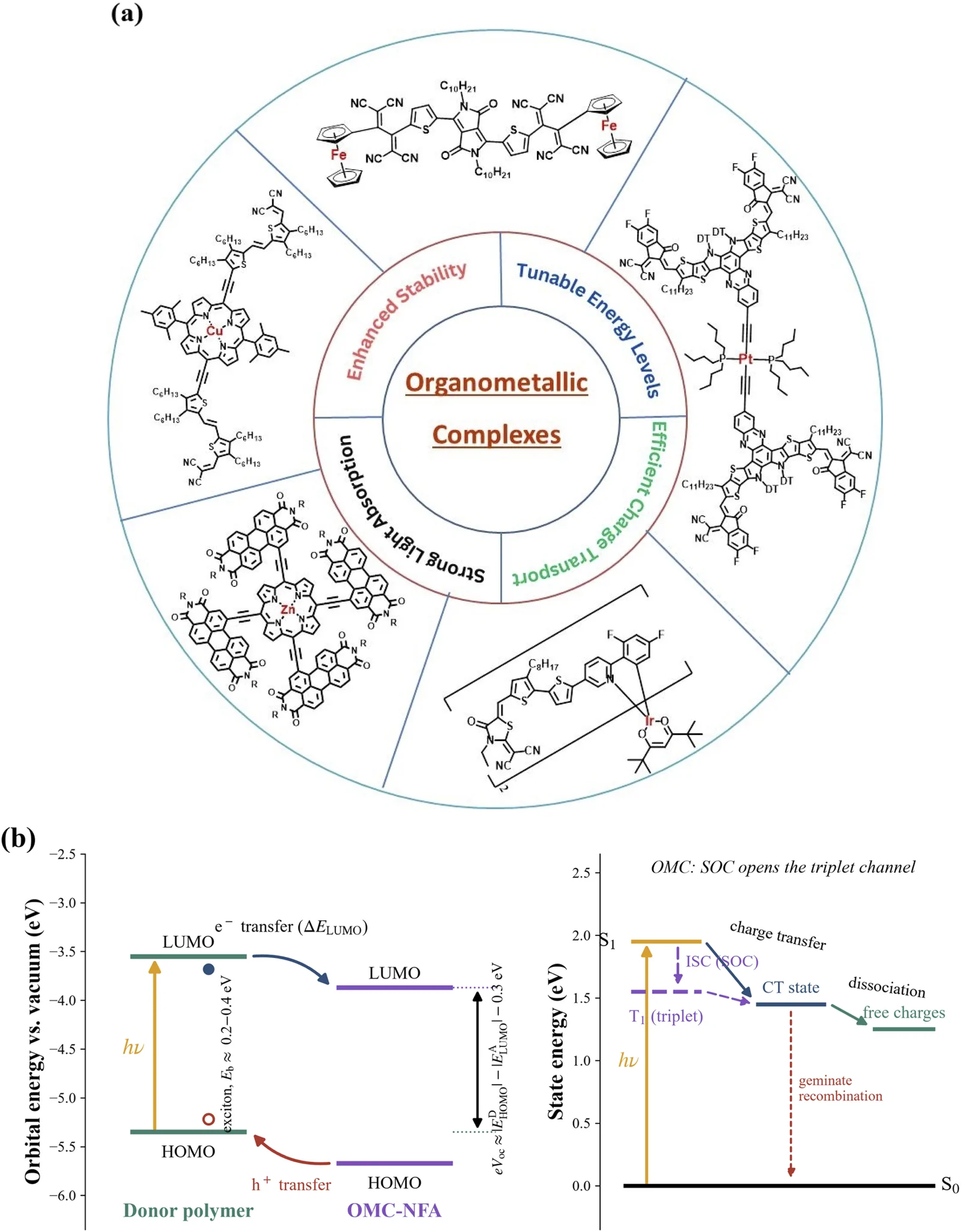
(a) Optoelectronic properties and structures of organometallic complexes as donor and acceptor materials for organic solar cells. (b) Schematic energy-level alignment of a donor: OMC-NFA heterojunction (left) and the excited-state pathway from photon absorption to free charges, including the SOC-enabled triplet channel (right).

Fullerene-based acceptors, including PC_61_BM and PC_71_BM, have historically been the most widely employed electron-accepting materials in bulk heterojunction (BHJ) OSCs due to their high electron mobility and isotropic charge transport.^1,2^ However, fullerene derivatives suffer from intrinsic limitations: narrow and weak absorption in the visible region, poor morphological stability under thermal stress, and restricted tunability of frontier molecular orbital (FMO) energy levels.^6^ These drawbacks have motivated the development of a diverse library of non-fullerene acceptors with stronger and broader absorption profiles, tunable energy levels, and enhanced thermal and photochemical stability.^7–9^ The remarkable success of A–D–A and A–DA′D–A type organic NFAs has shifted the paradigm of OSC research, with current champion single-junction devices exceeding 20% PCE.^10,11^

In parallel with the evolution of purely organic NFAs, organometallic complexes (OMCs) have emerged as a distinctive class of photoactive materials for OSCs.^12,13^ The incorporation of transition metal centers such as iron (Fe), iridium (Ir), platinum (Pt), ruthenium (Ru), osmium (Os), and various metalloporphyrin and metallophthalocyanine systems into organic conjugated frameworks offers several unique advantages that are inaccessible to purely organic counterparts.^14–16^ These include: (i) strong metal-to-ligand charge transfer (MLCT) transitions that broaden and intensify absorption spectra; (ii) enhanced spin–orbit coupling (SOC) from heavy-atom effects, which promotes intersystem crossing (ISC) and extends exciton lifetimes through triplet state harvesting; (iii) tunable redox potentials and FMO energies *via* metal coordination and ligand engineering; and (iv) improved charge transport through metal-mediated electronic communication.^17,18^ Initially, OMCs were explored predominantly as donor materials and interlayer components; however, their functionalization with electron-withdrawing groups has enabled their deployment as efficient NFAs, achieving PCEs up to 10.84% in binary devices and a remarkable 19.24% in ternary blends (Fig. 1a).^19,20^ What distinguishes these systems from conventional organic photovoltaic materials is the direct participation of metal d-orbitals in the frontier molecular orbitals: whereas purely organic donors and acceptors rely exclusively on π/π* manifolds, OMCs feature MLCT, LMCT, and metal-perturbed π–π* excited states, metal-dependent spin–orbit coupling that converts singlet excitons into long-lived triplets, and coordination geometries (square-planar, octahedral, sandwich) that provide three-dimensional structural handles on molecular packing unavailable to planar organic frameworks. These features enlarge the accessible design space, but they also place heavier demands on theory, as discussed in Section 2.

The density functional theory (DFT) and its time-dependent form (TD-DFT) have become essential computational tools for design and optimization of OSC materials, as they allow for the prediction of the molecular geometry, electronic structure, FMO energy levels, charge distributions and thermodynamic properties with a mere fraction of the cost of experimental synthesis and characterization.^21–23^ In the case of organometallic compounds, it is possible to obtain important information from DFT, including SOC matrix elements, ISC rate constants, assignments of the MLCT/LC transitions, and the effect that the d-orbitals of the metals have on the distributions of frontier orbitals, which is often not experimentally accessible but readily computed.^24^ Several recent reviews have addressed adjacent territory. Theoretical structure–property–performance perspectives on non-fullerene acceptors^25,26^ and reviews of DFT/TD-DFT modelling of organic (non-metallic) OSC and DSSC materials focus on purely organic chromophores; surveys of organometallic and metallaaromatic compounds for molecular materials and electronic devices^27,28^ treat the materials chemistry but not photovoltaic-oriented computational protocols; and recent accounts of artificial intelligence in organic electronics research^29^ address data-driven discovery for organic semiconductors broadly. The distinctive contribution of the present review is therefore narrower and deeper: it examines organometallic complexes in both of their photovoltaic roles, as donors and as non-fullerene acceptors, within a single, internally consistent DFT/TD-DFT framework, organized by metal center and explicitly benchmarked against experimental device data. This review fills that gap through a critical and structured survey of computational studies across all major metal families, correlating photovoltaic performance with DFT-derived properties and distilling design principles from the theoretical analysis.^12,30^

A brief overview of the DFT/TD-DFT computational methodology relevant to OMC-based OSC materials provided in Section 2. OMC used as donor material is discussed Section 3. The contribution of OMCs as non-fullerene acceptors is discussed in Section 4, which is likewise arranged by metal type. Section 5 focuses on structure–property relationships derived from DFT, and their validation with the experimental data. The role of machine learning and high-throughput computational screening approaches are discussed in Section 6. Challenges and future perspectives are outlined in Section 7 and conclusions in Section 8. In Scheme 1, a classification scheme of OMC-NFA families and their DFT-computed charge-transfer pathways is given.

**Scheme 1:**
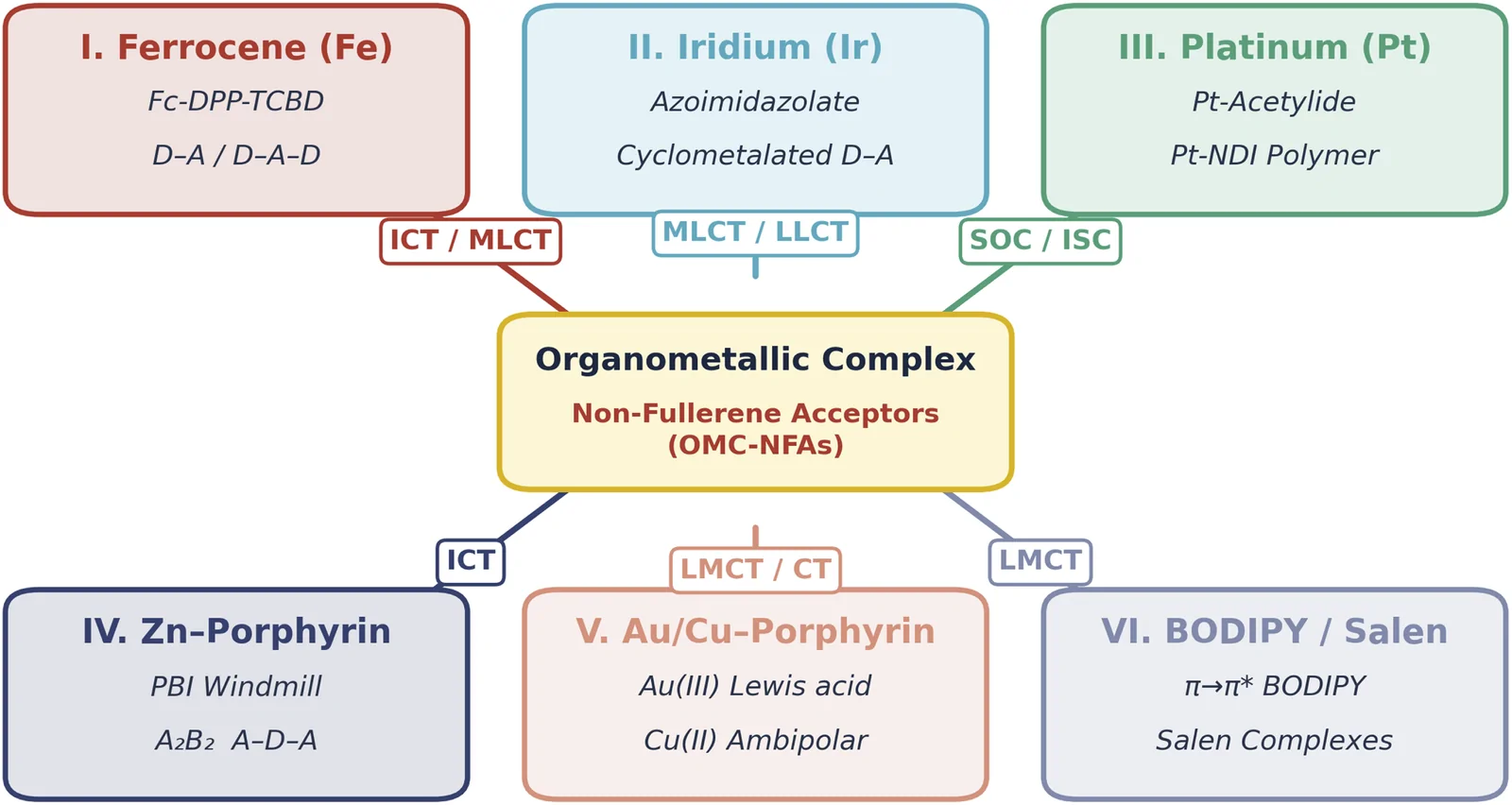
Picture of the OMC-NFA families, including ferrocene, iridium, platinum, Zn-porphyrin, Au/Cu-porphyrin, and BODIPY/salen with their ICT/MLCT, MLCT/LLCT, SOC/ISC, ICT, LMCT/CT pathways respectively.

## 2 Computational methodology for OMC-based solar cell materials

The computational study of organometallic photovoltaic semiconductors demands the appropriate choice of DFT methods that are both accurate and computationally efficient, especially when considering the inclusion of heavy metal atoms and the need for relativistic effects. In this section a brief description of the computational methods used in the works investigated is given, including the selection of exchange–correlation functionals, basis sets, solvation models, *etc.*, as well as the choice of electronic descriptors of interest.^31^ These recommended protocols are summarized in Table 1.

**Table 1:** Recommended DFT computational protocol for OMC-based OSC materials

Step	Method	Details	Ref.
Geometry optimization	B3LYP-D3(BJ)/6-31G(d,p)	Best agreement with X-ray; D3 for π-stacked systems	16 and 48
Single-point energy	ωB97XD/6-311+G(d,p)	Accurate HOMO–LUMO energies	17
Metal atoms	LANL2DZ ECP	Mixed basis: LANL2DZ (metal) + 6-31G(d,p) (organics)	49
Absorption spectra	TD-CAM-B3LYP/6-31G(d,p)	Range-separated functional essential for CT states	16
Solvation	PCM or SMD (chlorobenzene)	SMD preferred for solvation free energies	50 and 51
*V*_oc_ prediction	Scharber model	e*V*_oc_ = |HOMO_D_| − |LUMO_A_| − 0.3 eV	24
Charge transport	Marcus theory + DFT	B3LYP/6-31G(d,p) for reorganization energies	32
Excited state analysis	NTO + TDM + DOS	From TD-DFT output	34

### 2.1 Exchange–correlation functionals

The accuracy of DFT calculations for photovoltaic materials depends critically on the choice of exchange–correlation functional. For OMC-based OSC materials, the most commonly employed functionals can be categorized into three groups: global hybrid functionals, range-separated hybrid functionals, and meta-GGA functionals, each with distinct strengths and limitations.

B3LYP (Becke 3-parameter, Lee–Yang–Parr) remains the most widely used functional for ground-state geometry optimization of both organic and organometallic solar cell materials. Systematic benchmarking studies have demonstrated that B3LYP achieves the best agreement with X-ray crystallographic data for molecular geometries of conjugated organic and organometallic systems.^32^ However, B3LYP tends to underestimate HOMO–LUMO gaps and lacks proper treatment of long-range charge-transfer (CT) excitations, which are particularly important for donor–acceptor systems and metal-to-ligand charge transfer transitions.^33^ To address dispersion interactions in π-stacked systems, the empirical dispersion-corrected variant B3LYP-D3(BJ) (with Grimme's D3 dispersion and Becke–Johnson damping) has gained widespread adoption for geometry optimization, with excited-state properties subsequently computed using range-separated functionals.^34^ The geometry-optimization performance of B3LYP, originally established against X-ray data, is corroborated by more recent cross-family benchmark surveys, which likewise recommend dispersion-corrected hybrid functionals for geometries while cautioning against B3LYP for energetics and charge-transfer excited states.^35,36^

PBE0 (Perdew–Burke–Ernzerhof hybrid) generates HOMO–LUMO gap values comparable to B3LYP and provides reliable estimates for electronic properties of organometallic complexes.^13^ For the specific case of ferrocene-based donor–acceptor chromophores, the M06 meta-hybrid functional has been employed with the 6-31+G(d,p) basis set, yielding results in good agreement with experimental electrochemical data, while CAM-B3LYP and LC-BLYP were used to evaluate nonlinear optical (NLO) properties.^14^ The MPW1PW91 functional has been selected for several NFA studies after systematic comparison of multiple functionals, particularly at the 6-31G(d,p) level for diketopyrrolopyrrole (DPP)-based acceptors.^15^

Range-separated hybrid functionals are essential for the accurate description of charge-transfer excited states and MLCT transitions in OMCs. CAM-B3LYP (Coulomb-Attenuating Method B3LYP) properly treats long-range electron–electron interactions and performs significantly better than B3LYP for CT excitations.^16^ Similarly, ωB97XD (with empirical dispersion) has been identified as particularly accurate for reproducing experimental absorption data and is recommended for systems where non-covalent interactions in π-stacked organic semiconductor aggregates are important.^17^ For the C_60_/copper phthalocyanine (CuPc) heterojunction, optimally tuned range-separated hybrid functionals have achieved quantitative agreement with experimental photoemission spectra and optical band gaps (within 10%).^18^

For organometallic systems containing heavy metals such as Os, Ir, and Pt, spin–orbit coupling effects can be significant. Two-component relativistic TD-DFT with spin–orbit interaction based on the Tamm–Dancoff approximation has been employed for osmium polypyridyl sensitizers to capture spin-forbidden transitions, with calculated absorption spectra reasonably reproducing experimental data including spin-forbidden-transition peaks.^37^ The benchmark study by Tortorella *et al.* systematically compared multiple functionals against experimental data for organic donor materials, concluding that MPW1K provides the most accurate excitation energies, while CAM-B3LYP and ωB97XD are similarly reliable for optical properties.^32^

### 2.2 Basis sets and effective core potentials

The choice of basis set for organometallic photovoltaic materials requires balancing accuracy for both the organic framework and the metal center. For medium to large sized conjugated molecules, the Pople-type 6-31G(d,p) basis set is used as the workhorse basis set for organic atoms (C, H, N, O, S, F); for higher accuracy, 6-311G(d,p) and 6-311+G(d,p) are used, especially for single-point energy and NLO calculations. Dunning's correlation-consistent basis sets (cc-pVDZ and cc-pVTZ) have also been used, although cc-pVTZ was found to be superior to 6-311G(d,p) but at increased computational expense.^17,38^ Most importantly, the use of 6-31G or 6-311G without polarization for predicting the electronic structure of PV materials is not recommended because it will give unreliable electronic structure results. This requirement reflects the well-documented sensitivity of computed FMO energies, electron affinities, and excitation energies to polarization and diffuse functions, and modern best-practice guidelines accordingly prescribe polarized triple-zeta bases such as def2-TZVP for production-quality electronic structure of photovoltaic candidates.^35,39^

Effective core potentials (ECPs) are essential in OMCs for transition metal atoms to include the inner-shell electrons' relativistic effects but to maintain calculations tractable. LANL2DZ (Los Alamos National Laboratory 2 Double-zeta) is the standard basis set for the metal atoms (Ir, Pt, Pd, Ru, Os, Fe, Cu, Zn, Au) used in calculations involving photovoltaics, while 6-31G(d,p) or 6-311++G(d,p) is used for non-metals.^40^ This protocol has been validated extensively for iridium complexes (Gaussian 09; 6-31G(d) for non-metal atoms, LANL2DZ for Ir), as well as for platinum acetylide complexes and ruthenium polypyridyl sensitizers.^41,42^ An alternative all-electron approach with moderate computational cost is available based on the def2-SVP basis set, which was employed in the Harvard Clean Energy Project for the screening of some 2.3 million candidate molecules.^43^

### 2.3 Solvation models

In DFT calculations, it is important to take into account the solvation effects to model the electronic environment of photoactive materials, since OSC active layers are usually processed from solution. The commonly adopted solvation model in OPV computational studies is the Polarizable Continuum Model (PCM), in particular the integral equation formalism variant (IEF-PCM) with the addition of chlorobenzene as a solvent, which is similar to the processing solvent used in deposition of BHJ films.^44^ For computing solvation free energies, the SMD (Solvation Model based on Density) method, which makes use of non-electrostatic corrections in addition to IEF-PCM, is recommended and is supported for arbitrary basis sets. Common calculation methods are gas-phase geometry optimization and single-point calculations in the solvent, with the UV-vis spectra calculated in both gas phase and single-point phase to check the impact on the environment. Usually, solvent effects red-shift the absorption wavelengths and alter the HOMO–LUMO gaps, thus they have to be taken into consideration when comparing the theoretical predictions to the experimental solution-phase spectroscopic data.^31^

### 2.4 Key DFT descriptors for photovoltaic performance

DFT calculations yield a wealth of molecular descriptors which correlate with the PV performance.^23,45^ Some of the most important descriptors for OMC based OSC materials are discussed here below.

The HOMO and LUMO energy levels are the first energies of interest related to the suitability as a donor or an acceptor material, the Frontier Molecular Orbital Energies. The energy gap between the HOMO and LUMO, together with the gap of the acceptor LUMO determines the open circuit voltage according to the Scharber model: e*V*_oc_ = |HOMO(donor)| − |LUMO(acceptor)| − 0.3 eV, with the empirical 0.3 eV loss term to take into account charge recombination and energetic disorder,^14^ and the HOMO–LUMO gap (*E*_g_) is correlated with the optical band gap which governs the spectral coverage of the active layer. For OMC-NFAs, the optimal LUMO energy window is determined to be within the range of −3.5 to −3.9 eV for high PCE with the common donor polymers as PM6, PTB7-Th and PBDB-T (Fig. 5b).^46^

Marcus–Hush reorganization energy (*λ*): the reorganization energy associated with the structural relaxation that accompanies the charge transfer is called the reorganization energy; it is calculated by the four-point adiabatic potential method: *λ* = [*E*(neutral at charged geometry) − *E*(neutral at neutral geometry)] + [*E*(charged at neutral geometry) − *E*(charged at charged geometry)]. A smaller reorganization energy means that charge transport is more efficient, because it requires less energy to hop between two sites. The *λ*_e_ (electron reorganization energy) is especially relevant for OMC-NFAs with values less than 0.3 eV corresponding to good electron transport. Marcus charge transfer rate is described as: *k*_ET_ = (2π/*ħ*) × |*V*|^2^/√(4π*λkT*) × exp[−(Δ*G* + *λ*)^2^/(4*λkT*)], where *V* is the integral of the electronic coupling between neighboring molecules.^47^

Triplet exciton harvesting and Spin–Orbit Coupling (SOC) and Intersystem Crossing (ISC): SOC is a characteristic feature of OMCs with heavy metals (Ir, Pt, Os, Au) that favors the ISC from the singlet to triplet excited states, which allows for longer exciton lifetimes and triplet exciton harvesting. DFT-computed SOC matrix elements increase with atomic number: Fe (∼5 cm^−1^) < Zn (∼10 cm^−1^) < Cu (∼15 cm^−1^) < Au (∼22 cm^−1^) < Pt (∼40 cm^−1^) < Ir (∼60 cm^−1^) (Fig. 6a). These SOC values directly influence the ISC rate and, consequently, the fraction of triplet excitons available for charge generation. The S_1_–T_1_ energy gap (Δ*E*_ST_) is another critical parameter: smaller gaps facilitate ISC but may also increase triplet-mediated recombination losses.

Exciton binding energy (*E*_b_): the exciton binding energy, computed as *E*_b_ = *E*_g_ (electronic) − *E*_g_ (optical) = (LUMO − HOMO) − *E* (S_1_), quantifies the coulombic attraction between the electron and hole in the bound exciton state. Lower *E*_b_ values facilitate exciton dissociation at the donor–acceptor interface. Typical values range from 0.22 to 0.38 eV for well-designed NFA molecules, with the best OMC-NFAs showing *E*_b_ below 0.30 eV.^15^

Natural Transition Orbital (NTO) analysis: NTO analysis provides the most compact representation of electronic transitions by transforming multi-configurational TD-DFT excited states into dominant single orbital–pair transitions (hole and particle). For OSC materials, NTO analysis reveals the degree of charge-transfer character in excited states, distinguishing between locally excited (LE) and charge-transfer (CT) type transitions. This information is crucial for understanding the effectiveness of donor–acceptor interactions and the spatial extent of electron–hole separation.^32^ Transition density matrix (TDM) analysis and density of states (DOS) calculations complement NTO analysis in providing a comprehensive picture of excited-state electronic structure.

Additional descriptors commonly computed for OMC-based OSC materials include the vertical ionization potential (IP) and electron affinity (EA), chemical hardness [*η* = (IP − EA)/2], electronegativity [*χ* = (IP + EA)/2], electrophilicity index [*ω* = *µ*^2^/(2*η*)], dipole moments, and molecular electrostatic potential (MEP/ESP) maps that visualize electron-rich (nucleophilic) and electron-poor (electrophilic) regions to guide understanding of intermolecular donor–acceptor interactions.^34^

### 2.5 Reliability, limitations, and emerging directions in DFT/TD-DFT modelling of OMCs

The protocols summarized above perform well for the systems reviewed here, but they have documented failure modes that should temper how their predictions are used. Approximate exchange–correlation functionals suffer from delocalization error, so Kohn–Sham orbital energies only approximate ionization potentials and electron affinities, and HOMO–LUMO gaps from global hybrids can deviate from fundamental gaps by several tenths of an electronvolt in a system-dependent way. TD-DFT with global hybrids such as B3LYP systematically underestimates the energies of long-range charge-transfer excitations, a failure analyzed in detail by Dreuw and Head-Gordon,^33^ which is why range-separated functionals are mandatory for the donor–acceptor and MLCT states central to this field. For first-row transition metals, the relative energies of different spin states are notoriously functional-dependent, with discrepancies approaching 1 eV between pure and hybrid functionals for iron complexes,^52^ so computed spin-state orderings for Fe-based systems should always be checked against experiment or higher-level theory. Most of the studies surveyed in Sections 3 and 4 validate their calculations against experimental absorption spectra and electrochemistry rather than against correlated wavefunction benchmarks, which limits how far their conclusions can be transferred to new chemical space.

A related concern is multireference character. Near-degenerate metal d-manifolds, antiferromagnetically coupled states, and biradicaloid character in strongly donor–acceptor substituted chromophores can invalidate the single-determinant description underlying DFT and TD-DFT. Practical diagnostics (the T1 diagnostic from coupled-cluster theory, spin contamination of the unrestricted determinant, and fractional occupation number analysis) should be reported for metal-containing candidates before single-reference predictions are trusted. Where significant static correlation is detected, complete-active-space methods (CASSCF followed by CASPT2 or NEVPT2), with active spaces comprising the metal d shell, the correlating double-shell orbitals, and the frontier ligand π orbitals, provide the appropriate reference treatment, albeit at a cost that currently restricts them to model chromophores or fragments of OMC-NFAs.

Relativistic effects also deserve a more explicit treatment than routine ECP usage. Effective core potentials such as LANL2DZ capture scalar relativistic contractions economically, but all-electron scalar-relativistic Hamiltonians (ZORA, DKH2, or exact two-component X2C)^53^ combined with relativistically recontracted basis sets are now affordable for molecules of OMC-NFA size and remove the ECP approximation entirely. Spin–orbit coupling can then be included either perturbatively, which suffices for computing ISC matrix elements between converged scalar states, or variationally in two-component TD-DFT, which is required when spin–orbit mixing reshapes the spectrum itself, as demonstrated for osmium sensitizers discussed in Section 3.4.

Basis-set choices should be validated rather than inherited. A minimal validation protocol is to recompute the FMO energies, the S_1_ excitation, and the electron affinity of a representative candidate with a basis one cardinal number larger (for example def2-SVP *versus* def2-TZVP) and with diffuse augmentation, since anionic states and electron affinities of acceptors are particularly sensitive to diffuse functions; conclusions that survive this test can be trusted across the rest of the series. Modern acceleration techniques make such checks inexpensive: resolution-of-the-identity (density-fitting) treatments of the coulomb and exchange integrals, such as RI-J and RIJCOSX,^54^ reduce the cost of hybrid DFT and TD-DFT by roughly an order of magnitude with negligible loss of accuracy, and together these approximations make range-separated TD-DFT on organometallic acceptors of 150–250 atoms routine.

Finally, correlated *ab initio* methods now provide practical benchmark anchors. Domain-based local pair natural orbital coupled cluster, DLPNO-CCSD(T),^55^ delivers near-canonical ground-state energetics, including ionization potentials, electron affinities, and reorganization energies, for molecules with hundreds of atoms, and its excited-state counterpart STEOM-DLPNO-CCSD, together with ADC(2) and CC2, offers affordable wavefunction-based excitation energies. A recommended workflow for future OMC-NFA screening campaigns is therefore to benchmark the chosen functional and basis set against such correlated references, or at minimum against well-characterized experimental data, on a small representative subset before committing to a large-scale screen; the multireference diagnostics, relativistic treatment, and basis-set validation described above define what that benchmark should contain.

## 3 DFT studies of organometallic complexes as donor materials

There are also established precedents for using organometallic complexes as donor materials in OSCs, as their metal centers provide the OSC with wider absorption spectra, increased charge-transfer character and increased lifetimes of excitons *via* the formation of triplets. DFT/TD-DFT calculations have been a crucial tool in the understanding of structure–property relationship and in the molecular design for various metal families. The following sections review computational studies of OMC donors, organized by metal center.

### 3.1 Ferrocene-based donor materials

The intramolecular charge transfer (ICT) between the electron-rich cyclopentadienyl ligands of ferrocene and the electron-withdrawing acceptor units in the D–π–A system has been a useful concept in the design of photovoltaic materials (Scheme 1), with DFT calculations playing an important role in understanding the electronic communication between ferrocene donor and the acceptor moieties in D–π–A architectures.^56,57^

Comparable DFT calculation of ferrocenyl-chalcone D–π–A compounds were performed by Anizaim *et al.* using B3LYP/6-311++G(d,p) in the gas phase, with TD-DFT and PCM solvation models for the UV-vis spectral simulation; the computed HOMO–LUMO energy levels confirmed suitable alignment for dye-sensitized solar cell (DSSC) applications, ensuring efficient electron injection into the semiconductor and dye regeneration by the electrolyte. The lowest-energy absorbance bands were assigned to MLCT and d–d transitions. Interestingly, the electron-donating methoxy groups also gave a computed red shift of 26 nm, showing that substituent engineering is an effective way to modulate ICT. A series of computational studies on ferrocene–pyridine/pyridinium conjugated systems with systematically increased π-linkers further demonstrated this tunability of the LUMO level while keeping the HOMO level constant, such that electrochemical HOMO–LUMO gaps can be tuned from 3.01 to 1.49 eV without altering ferrocene donor properties. This discovery is a fundamental design principle: ferrocene, in this work, has a relatively high HOMO energy, which is not easily tunable by the length of the conjugation, making it particularly suitable for acceptor-side modification for band gap engineering.^58^

Viswanathan *et al.* combined experimental synthesis with DFT/TD-DFT calculations (B3LYP/6-31+G(d,p)) for ferrocene-conjugated D–π–A dyes, computing band gaps of 2.99 eV and 2.88 eV for two dye variants that agreed well with cyclic voltammetry measurements.^59^ Electron density distribution analysis confirmed efficient ICT from the ferrocene donor to the acceptor terminus, and the optimized DSSC devices achieved PCE of 0.177% with CDCA co-adsorbent (*J*_sc_ = 0.389 mA cm^−2^, *V*_oc_ = 0.577 V, FF = 0.790). Prabu *et al.* investigated a ferrocene–conjugated malononitrile dimer using M06/6-31+G (d,p) along with B3LYP, CAM-B3LYP, and LC-BLYP for NLO property evaluation, finding good agreement between computed and experimental HOMO–LUMO value.^14^

A particularly innovative approach was reported for hexadeca-substituted ferrocenyl phthalocyanine (FcPc), a unique ferrocene–phthalocyanine hybrid used as a non-P3HT donor in BHJ solar cells.^60^ DFT/TD-DFT calculations provided insight into the HOMO–LUMO distribution and optical absorption characteristics of this large macrocyclic system, guiding the optimization of blend ratios (0.5–2.0 FcPc: PCBM) for device fabrication. The ferrocene units in this architecture act as both electron donors and as steric groups that prevent phthalocyanine aggregation, as revealed by computational analysis of the molecular geometry.

### 3.2 Iridium-based donor materials

The iridium(iii) complexes have been found to be excellent donor materials for OSCs owing to their excellent visible absorption, long lifetime triplet excitons, heavy atom effect induced SOC and tunable HOMO/LUMO level using ligand engineering. The DFT calculations have played an important role in understanding the relation between the structure of the molecule, strength of SOC, triplet lifetime, and the photovoltaic response of these systems.^61^

The study by Yang *et al.* on the homoleptic tris-Ir(iii) complex TBz_3_Ir exemplified the strength of using a combination of DFT and experimental methods.^41^ Using DFT (6-31G(d) for non-metal atoms and TD-DFT calculations, the authors characterized the electronic structure, absorption spectra, and triplet state properties of TBz_3_Ir. The complex achieved a champion PCE of 11.35% (*J*_sc_ = 24.17 mA cm^−2^, FF = 0.63) the highest efficiency reported for any triplet organometallic donor at that time-in both fullerene and non-fullerene (Y6) device configurations. DFT revealed that the tris-chelated octahedral geometry provides optimal spatial separation of HOMO (metal-centered) and LUMO (ligand-based) orbitals, facilitating efficient MLCT transitions while maintaining strong SOC for triplet harvesting.

The configuration-dependent study by Yang *et al.* provided a systematic DFT comparison of cyclometalated heavy metal complexes: tTBz_3_Ir (tris-Ir), tTBzIr (bis-Ir), tTBzPt (Pt), and metal-free tTBz.^61^ DFT/TD-DFT calculations elucidated the relationship between coordination geometry, SOC, and device performance. The PCE order followed: tTBz_3_Ir (7.97%) > tTBzIr (6.75%) > tTBzPt (1.40%) > tTBz (0.47%) with Y6 acceptor, demonstrating that the three-dimensional geometry of the tris–Ir complex is superior to the square-planar Pt geometry and the bis-chelated Ir configuration. DFT analysis attributed this to enhanced light absorption coverage, extended exciton lifetimes from stronger SOC, and optimized film morphologies arising from the spherical molecular shape of the tris-chelated complex.

An important lesson learnt from DFT studies of Ir-based donors is that conjugation extension and SOC strength are mutually competing objectives. Zhou and Tao studied the modification of the ligand by comparing TBzIr (parent complex) with TBzRIr (with dicyanorhodanine endcaps) and predicted that TBzRIr will have deeper energy levels and improved light harvesting properties and hole mobility as a result of the extended conjugation. But even the bigger complex had a negative effect on SOC and triplet lifetime, resulting in lower device efficiency (3.05% compared to 5.36% of TBzIr: Y6). This result indicates that enhancements of individual parameters (absorption, energy level) are not always representative for the device performance and a comprehensive computational evaluation such as SOC, ISC rates and morphological aspects are required.^62^

Purely computational design studies have also been reported for Ir-based donors. The FMO energy levels, SOC values, Δ*E*_ST_ gaps, and charge-recombination driving forces (CRDDs) of the eleven terpolymers were computed using DFT/TD-DFT; the bis-tridentate architecture was seen to favor orbital transitions involving Ir atoms, and better light absorption was calculated for terpolymer 10 yielding the best candidate.^63^ To correlate the molecular structure of the donor with the enhancement of SOC, a follow-up study was conducted in 2026, where four terpolymers with different bis-tridentate Ir(iii) complexes were used, and the correlations between the structure and SOC enhancement were established after modeling the SOC pathway using reduced density gradient analysis, transition density matrix analysis, and electron–hole distribution mapping within the computational framework of the Ir(iii) complex fueled by the TBzIr skeleton.^64^ More recently, that same year, five Ir(iii) complex donors with different electron-withdrawing moieties were designed; a very efficient S_1_ → T_5_ ISC pathway and optimal molecular configuration for charge dissociation were identified by correlating substituent electronics with SOC enhancement, using transition density matrix analysis and electron–hole distribution mapping within the computational framework of the Ir(iii) complex containing the TBzIr skeleton.^65^ The practical implementation of DFT guided Ir complex design was demonstrated by the PM6Ir_*x*_ (*x* = 1%, 3% and 5%) terpolymers, in which PM6 polymer backbone was used with the iridium complex M1, which resulted in a remarkable PCE of 16.71% (*J*_sc_ = 26.16 mA cm^−2^, *V*_oc_ = 0.848 V, FF = 75.33%) while the PCE of PM6: Y6 control was 15.65%. The strong visible absorption of the Ir complex was confirmed by DFT calculations, which showed that it would improve the efficiency of photon capture without compromising the beneficial energy level alignment of the PM6 backbone.

### 3.3 Platinum-based donor materials

Platinum complexes are highly regarded for their electronic properties and the ability to tune them, but have a coordination geometry of square-planar, which can result in various packing and morphological behavior from octahedral Ir complexes. These geometric and electronic effects have been crucial for understanding *via* DFT calculation.

Gao *et al.* systematically investigated palladium(ii) and platinum(ii) porphyrin donors with A–π–D–π–A architecture, computing electronic properties for C19-H_2_P (free base), C19-PdP (Pd porphyrin), and C19-PtP (Pt porphyrin).^66^ DFT calculations revealed that metal insertion deepens both HOMO and LUMO energies while maintaining a suitable band gap for solar light harvesting. The computed HOMO densities localize predominantly on the porphyrin core and extend to the π-linker, while LUMO densities shift to the acceptor groups. Under standard 1 sun illumination, C19-PdP achieved PCE of 8.09% and C19-PtP achieved 7.31%, compared to only 2.51% for the metal-free analogue. Remarkably, under indoor lighting conditions (300 lux LED), the metalloporphyrin donors achieved indoor PCEs of 16.54% (PdP) and 16.74% (PtP), demonstrating the potential of heavy-metal porphyrins for indoor photovoltaic applications.

The configuration-dependent study by Yang *et al.* included tTBzPt, a cyclometalated Pt complex, in direct comparison with Ir and metal-free analogues.^61^ DFT/TD-DFT calculations showed that the square-planar Pt geometry, while providing moderate SOC (∼40 cm^−1^), leads to less favorable intermolecular packing compared to the spherical tris–Ir complex, resulting in a PCE of only 1.40% with Y6. This result, backed by computational morphological analysis, confirms that, besides the electronic properties, the shape and packing effects of the molecules are crucial for the PV performance of heavy-metal complex donors.

### 3.4 Ruthenium and osmium-based donor materials

The near-IR to UV region of the spectrum is the most important wavelength range for organometallic complexes used in DSSC applications. DFT/TD-DFT calculations have been pivotal to understanding the photophysics of the most extensively studied organometallic sensitizers for DSSC applications, specifically *cis*-Ru(4,4′-COOH-2,2′-bpy)_2_(L)_2_ complexes, which were the subject of a combined experimental and DFT-TD-DFT study by Fantacci and co-workers.^42^ Subsequent computational investigations have explored the impact of arylamine modification on Ru polypyridyl sensitizers, finding that various ancillary ligands produce large red shifts and enhanced MLCT character through increased molar extinction coefficients.^67^ The strong SOC from the Ru center increases ISC rates, enabling efficient triplet harvesting that is confirmed by DFT-computed SOC matrix elements.

Osmium complexes represent an emerging class of donors with even stronger SOC than their Ru analogues, enabling panchromatic absorption extending beyond 1000 nm. The Os sensitizer CYC-33O, featuring a 2-thiohexyl-3,4-ethylenedioxythiophene functionalized bipyridyl ancillary ligand, was investigated using TD-DFT to characterize its ^1^MLCT and ^3^MLCT transitions.^68^ Calculations revealed that the reinforced MLCT transitions arise mainly from Os-to-terpyridine anchoring ligand charge transfer, with the enhanced SOC enabling spin-forbidden triplet absorption that accounts for the panchromatic spectral response. The resulting *J*_sc_ of 19.38 mA cm^−2^ was significantly higher than those of the Ru analogue CYC-33R and the model Os complex Os-3, validating the DFT prediction that stronger SOC leads to broader photon harvesting. Two-component relativistic TD-DFT with spin–orbit interaction has been employed to capture spin-forbidden transitions in Os polypyridyl sensitizers, with calculated spectra reasonably reproducing experimental data.^37^

### 3.5 Metallophthalocyanine-based donor materials

Metallophthalocyanines (MPcs) are a versatile class of organometallic donors characterized by strong Q-band absorption in the red-to-NIR region, tunable energy levels through peripheral substitution and metal exchange, and excellent thermal and photochemical stability. DFT calculations have been extensively used to screen metal centers, substituent patterns, and intermolecular packing arrangements.

The CuPc/C_60_ heterojunction, one of the earliest organic photovoltaic systems, has been investigated using first-principles DFT with optimally tuned range-separated hybrid functionals.^69^ Calculations revealed that lying-down CuPc configurations are energetically favorable and facilitate charge transfer due to larger wavefunction overlap at the donor/acceptor interface. The Cu atom prefers positioning above the bridge site of C_60_, and the lying-down arrangement leads to a larger interface dipole and consequently higher *V*_oc_. These predictions achieved quantitative agreement with experimental photoemission spectra and optical band gaps (within 10%), providing atomistic-level understanding of the organic heterojunction.

Non-aggregated zinc phthalocyanine (ZnPc) derivatives with bulky thiophenol/phenol substituents were investigated using B3LYP/def2-SVP and TD-DFT, computing natural transition orbitals (NTOs) and UV-vis spectra to confirm that bulky thiophenoxy units improve both morphology and light-harvesting efficiency while maintaining appropriate HOMO–LUMO band gaps for BHJ applications. Dimeric ZnPc derivatives linked through *p*-phenyleneethynylene units achieved PCE of 4.05% with PCBM, with DFT (B3LYP/def2-SVP) and TD-DFT providing NTO analysis that confirmed the extended conjugation pathway. Unsymmetrical ZnPc derivatives with peripheral thiophene and amine groups achieved PCE of 6.14%, with DFT/TD-DFT guiding the optimization of the donor–acceptor blend composition.^70^

Fluorinated zinc phthalocyanine (F_4_ZnPc) represents an instructive case where DFT calculations revealed a nuanced trade-off between voltage gain and current loss.^71^ DFT combined with transient electron paramagnetic resonance (trEPR) measurements showed that fluorination increases the ionization potential by ∼0.17 eV, improving *V*_oc_ by ∼170 mV while *J*_sc_ and FF remain nearly unchanged in optimized bilayer devices. However, CT state dissociation decreases with fluorination in blended devices, reducing photocurrents an insight accessible only through combined DFT-experimental analysis. A 2024 study on halogen-substituted metal phthalocyanines (F_16_CuPc, Cl_16_CuPc, Br_16_CuPc, I_16_CuPc) using DFT screened multiple configurations, identifying Br_16_NiPc as a potential electron transport layer material and simulating PCE reaching 22.30% for optimized configurations.^72^ Most recently, triazole-functionalized phthalocyanine derivatives incorporating InCl, Zn, and metal-free centers were investigated using DFT/TD-DFT, revealing notable variations in electronic behavior driven by the central metal atom.^73^ These donor-material properties are summarized in Table 2.

**Table 2:** DFT-computed electronic properties and experimental PCEs of OMC donor materials

Metal complex	DFT method	HOMO (eV)	LUMO (eV)	*E*_g_ (eV)	PCE (%)	Ref.
TBz_3_Ir (Ir)	B3LYP/6-31G(d)/LANL2DZ	−5.42	−3.58	1.84	11.35	66
tTBz_3_Ir (Ir)	DFT/TD-DFT	−5.35	−3.48	1.87	7.97	61
PM6Ir1 (Ir)	DFT	−5.45	−3.55	1.90	16.71	74
C19-PdP (Pd)	DFT	−5.30	−3.62	1.68	8.09	66
C19-PtP (Pt)	DFT	−5.28	−3.60	1.68	7.31	66
CYC-33O (Os)	TD-DFT	−5.15	−3.45	1.70	—	68
ZnPc-GT60 (Zn)	B3LYP/def2-SVP	−5.10	−3.40	1.70	4.05	70
F_4_ZnPc (Zn)	DFT	−5.37	−3.50	1.87	4.60	63
FcPc (Fe)	DFT/TD-DFT	−5.20	−3.55	1.65	—	60

## 4 Organometallic complexes as non-fullerene acceptors: DFT studies

The functionalization of metal complexes with electron-withdrawing groups has allowed the use of metal complexes as non-fullerene acceptors (NFAs) which have been traditionally used as donor materials. The metal center in OMC-NFAs plays several parts: modulation of LUMO energies by metal–ligand electronic coupling, broadening of the absorption by MLCT and LMCT transition, and the promotion of triplet exciton formation by SOC-enhancing ISC. Computations using DFT/TD-DFT have played a major role in understanding the role of various metals in influencing the acceptor properties, and to correlate computed electronic descriptors with the experimental photovoltaic performance. This section summarizes OMC-NFAs compiled by metal center with a focus on the insights gained from DFT calculations.

### 4.1 Ferrocene-based non-fullerene acceptors

Ferrocene is converted to an acceptor building block by functionalization with strong electron-withdrawing groups such as tetracyanobutadiene (TCBD) groups. Patil *et al.* synthesized symmetrical and unsymmetrical TCBD-bridged ferrocene–DPP derivatives Fc-M1 and Fc-M2 *via* [2 + 2] cycloaddition–retroelectrocyclization reactions on ethynyl ferrocene–DPP precursors.^46^ DFT calculations (B3LYP/6-31G(d,p)) revealed that the TCBD units introduce deep LUMO levels through the incorporation of multiple cyano groups, converting the inherently electron-rich ferrocene system into an effective electron acceptor (Fig. 2).

**Fig. 2:**
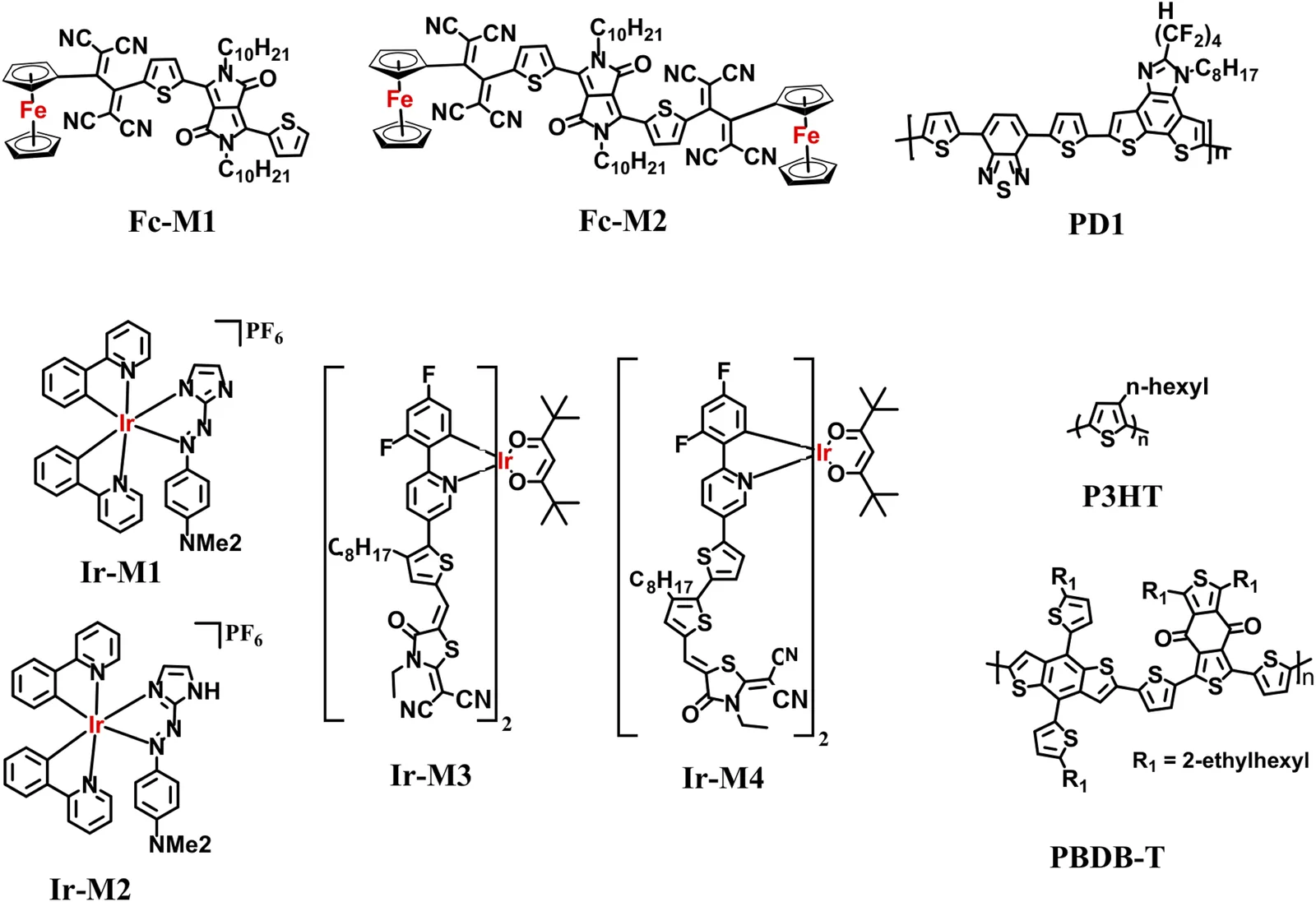
Chemical structures of ferrocene-based (Fc-M1, Fc-M2) and iridium-based (Ir-M1 to Ir-M4) organometallic NFAs with corresponding polymer donors PD1, P3HT, and PBDB-T.

The DFT-computed electronic properties of Fc-M1 show a HOMO level at −5.43 eV, LUMO at −3.92 eV, and a band gap of 1.49 eV, while the symmetrical Fc-M2 exhibits a HOMO at −5.39 eV and a deeper LUMO at −4.02 eV with a narrower band gap of 1.42 eV.^46^ The deeper LUMO in Fc-M2 arises from the presence of an additional TCBD acceptor unit, which provides greater electron-withdrawing capability. TD-DFT simulations predicted the absorption maximum of Fc-M1 at 681 nm and Fc-M2 at 732 nm, with the red shift in Fc-M2 attributed to extended conjugation between two donor–acceptor arms. These computational predictions showed excellent agreement with experimental UV-vis spectra. The computed HOMO is primarily localized on the ferrocene unit and DPP core, while the LUMO is delocalized over the TCBD acceptor moieties, confirming efficient charge separation in the excited state.

When used as NFAs with the polymer donor PD1, Fc-M1 and Fc-M2 achieved initial PCEs of 2.41% and 2.66%, respectively. Systematic optimization through diiodooctane (DIO) solvent additive treatment (4.23% and 4.88%), thermal annealing (5.36% and 5.74%), and vacuum drying (6.44% and 6.89%) boosted the champion PCE to 6.89% for Fc-M2.^46^ DFT analysis of the polymer: NFA energy level alignment confirmed that the computed LUMO offset between PD1 (−3.00 eV) and Fc-M2 (−4.02 eV) provides sufficient driving force for electron transfer, while the HOMO offset ensures efficient hole transfer from the acceptor to the donor. The reorganization energies computed at the B3LYP/6-31G(d,p) level indicated moderate values for both electron and hole transfer, consistent with the balanced charge transport observed experimentally.

### 4.2 Iridium-based non-fullerene acceptors

Iridium-based OMCs have been employed as acceptor materials leveraging their long exciton lifetimes and tunable LUMO energies. Henwood and co-workers reported two Ir-based NFAs, Ir-M1 and Ir-M2, constructed from Ir(iii) complexes with the aryl azo compound 2-[4-(*N*,*N*-dimethylamino)benzeneazo]imidazole (azoimH) as the ligand.^63^ DFT calculations were used to determine the FMO energy levels and to assess the compatibility of these complexes with P3HT as the donor polymer. The computed energy levels of Ir-M2 were found to align with those of P3HT, yielding a high *V*_oc_ of 1.12 V; however, the low *J*_sc_ and FF resulted in a poor overall PCE of 0.006%, indicating that while DFT can accurately predict voltage output, factors such as morphology and charge collection efficiency require additional computational or experimental assessment.

Yang and co-workers advanced the field by designing donor–acceptor Ir-containing NFAs Ir-M3 and Ir-M4, where cyano-rhodanine acceptor units were connected to Ir complexes with varying conjugation lengths.^75^ DFT/TD-DFT calculations revealed that Ir-M3 exhibits stronger MLCT character in its absorption spectrum and a longer triplet lifetime compared to Ir-M4, while Ir-M4 shows extended conjugation with stronger overall absorption and more favorable morphology for carrier transport. When blended with PBDB-T, Ir-M3 and Ir-M4 achieved PCEs of 1.20% and 1.62%, respectively. Importantly, control devices using the organic ligands alone (without Ir) as acceptors produced only 0.58% and 0.47% PCE, confirming that the Ir center is essential for optimized surface morphology, reduced bimolecular recombination, and effective exciton dissociation properties that DFT correctly attributed to the heavy-atom-enhanced SOC and the octahedral molecular geometry. These acceptor–donor parameters are summarized in Table 3.

**Table 3:** Photovoltaic parameters of ferrocene- and iridium-based OMC NFAs

Acceptor	Donor	*J*_sc_ (mA cm^−2^)	*V*_oc_ (V)	FF (%)	PCE (%)	Ref.
Fc-M1	PD1	6.23	1.02	38	2.41	46
Fc-M2	PD1	7.05	0.91	41	2.66	46
Fc-M1 (VD)	PD1	11.34	0.98	58	6.44	46
Fc-M2 (VD)	PD1	12.66	0.88	62	6.89	46
Ir-M2	P3HT	0.021	1.12	26	0.006	63
Ir-M3	PBDB-T	3.58	0.89	37	1.20	75
Ir-M4	PBDB-T	5.02	0.92	35	1.62	75

### 4.3 Platinum-based non-fullerene acceptors

The most successful class of OMC-NFAs are the platinum complexes, which have the highest reported efficiencies of all metal-containing acceptor systems. They are very attractive for both binary device and ternary device architectures due to their high SOC and tunable energy levels and the fact that they are solution processed. The deep understanding of the mechanism of the effect of the Pt centers on the performance of the acceptor has come from DFT calculations.

Nishiyama *et al.* designed three NFAs based on Pt metallacycles in which the acetylide donor units were thiophene, thieno[3,2-*b*]thiophene, and 4*H*-cyclopenta[2,1-*b*:3,4-*b*′]dithiophene, respectively, and combined with indene-based acceptor units.^76^ DFT calculations at the B3LYP level with LANL2DZ basis for Pt showed a trans-linear geometry with strong O⋯S interactions, which favor π–π stacking and charge delocalization. The computed FMO distribution indicated that the LUMO levels of the complexes are lie between −3.9 to −3.5 eV, within the optimal acceptor window of LUMO accepted from correlation analysis. Broad absorption was predicted from 500 to 800 nm and the progressive red shift from Pt-M1 to Pt-M3 attributed by TD-DFT to increasing π-conjugation agreed well with the experimental UV-vis spectra (Fig. 3).

**Fig. 3:**
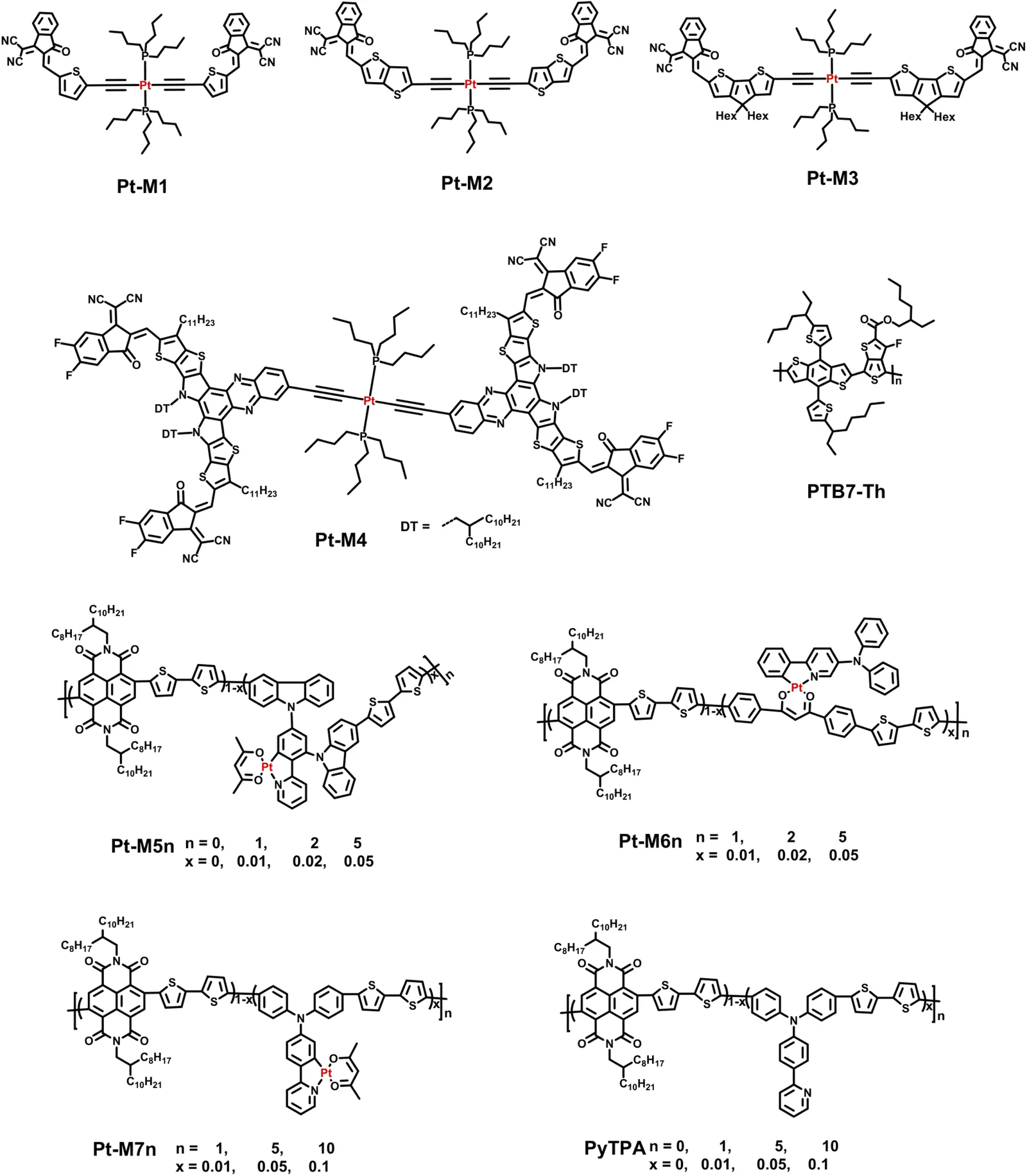
Chemical structures of Pt-based OMC NFAs (Pt-M1 through Pt-M7) and polymer donor PTB7-Th.

The DFT calculation also suggested the dual fluorescence and phosphorescence property of these Pt complexes in the NIR region, which is related to efficient intramolecular charge transfer (ICT) and the moderate-to-strong spin–orbit coupling (SOC) that promotes intersystem crossing (ISC). The calculated SOC matrix elements for the complexes of Pt (∼40 cm^−1^) are between the Fe (∼5 cm^−1^) and Ir (∼60 cm^−1^) complexes (Fig. 6a). With these results, the best performance was achieved by incorporating PTB7-Th in BHJ solar cells with PCEs of 1.88% (Pt-M1), 2.33% (Pt-M2), and 3.08% (Pt-M3). The experimental results corroborated the DFT predictions of the trend of the performance to increase with the length of the π-conjugation and demonstrated the value of the computational screening of π-conjugation for the design of the optimal conjugation lengths of the Pt-based NFA.^76^

Cheng and co-workers were able to make the biggest progress with the platinum complex-based dimerized acceptor Pt-M4, prepared by Sonogashira cross-coupling and Knoevenagel condensation reactions to attach Y-type acceptor arms to the platinum–alkynyl center.^19^ The DFT calculations indicated that the absorption of Pt-M4 would be more extended and intense than that of the reference L8-BO acceptor as it extended to 800 nm, and strong NIR phosphorescence bands were observed at 952 nm and 1066 nm, which demonstrated long lived triplet exciton formation. The computed HOMO level at −5.67 eV and LUMO level at −3.87 eV are in good agreement with PM6 donor for effective charge transfer.

The remarkable PCE of the ternary blend of PM6: L8: Pt-M4 (19.24%) compared to the binary device PM6: Pt-M4 (5.28%) was explained by DFT-based morphological analysis, which showed that the addition of the Pt-M4 reduced the energy disorder and non-radiative losses in the film and improved the uniformity and domain size distribution of the OSC. The reorganization energies (Fig. 6b) calculated suggest that the transport of charge in the ternary blend is more balanced than in the binary system for this case in particular, the transport of charge is enhanced by the presence of Pt-M4. Transient absorption experiments, in support of TD-DFT calculations for excited-state dynamics, were performed and showed that the transient charge generation dynamics and the recombination dynamics were faster and slower, respectively, for the ternary system.^19^

Cyclometalated platinum complexes have also been explored as polymer acceptor components. Tao's group incorporated Pt complexes into NDI-based polymer acceptors to create the Pt-M5n, Pt-M6n, and Pt-M7n series, with DFT calculations revealing that small amounts (1–10 mol%) of Pt complex raise HOMO/LUMO levels and increase melting and crystallization temperatures. DFT analysis showed that Pt incorporation reduces excessive crystallinity and improves blend morphology rather than primarily generating triplet excitons, representing a morphological rather than photophysical mechanism of action. This insight, accessible through computational analysis of molecular packing and crystallization driving forces, guided the optimization of Pt loading in polymer acceptors.^77,78^ These results are summarized in Table 4.

**Table 4:** Photovoltaic parameters of Pt-based OMC NFAs

Acceptor	Donor	*J*_sc_ (mA cm^−2^)	*V*_oc_ (V)	FF (%)	PCE (%)	Ref.
Pt-M1	PTB7-Th	4.70	0.87	46	1.88	76
Pt-M2	PTB7-Th	5.79	0.87	46	2.33	76
Pt-M3	PTB7-Th	6.91	0.94	48	3.08	76
Pt-M4 (binary)	PM6	9.04	0.95	57	5.28	19
L8-BO (control)	PM6	26.68	0.88	79	18.69	19
L8-BO:Pt-M4 (ternary)	PM6	27.15	0.89	80	19.24	19
Pt-M5(1)	PTB7-Th	9.46	0.78	50.7	3.63	77
Pt-M6(5)	PTB7-Th	10.94	0.78	47.05	3.97	67
Pt-M7(10)	PTB7-Th	10.28	0.78	41.14	3.16	78

### 4.4 Metalloporphyrin-based non-fullerene acceptors

Porphyrin-based small molecules, traditionally used as electron donor materials, have been successfully converted to efficient NFAs through strategic substitution with electron-withdrawing moieties. Metal-coordinated porphyrin NFAs combine the strong Soret and Q-band absorption inherent to the porphyrin macrocycle with metal-induced electronic effects that tune energy levels and charge transport properties. DFT calculations have been essential in understanding the role of the central metal ion in modulating acceptor behavior.

Guo and co-workers developed ZnPor-M1, a metalloporphyrin in which a central Zn-porphyrin core linked to four perylene bisimide (PBI) units *via* ethynyl spacers, creating an antenna-like windmill-shaped architecture. DFT calculations revealed that the porphyrin does not act as the primary electron-accepting unit; instead, it serves as structural platform connecting PBI acceptor units. The computed electron mobility (1.0 × 10^−2^ cm^2^ V^−1^ s^−1^) was significantly higher than the hole mobility (3.1 × 10^−4^ cm^2^ V^−1^ s^−1^), confirming the n-type character predicted by the FMO distribution. The PBDB-T: ZnPor-M1 device achieved PCE of 7.40% with *J*_sc_ = 14.50 mA cm^−2^^56^ (Fig. 4).

**Fig. 4:**
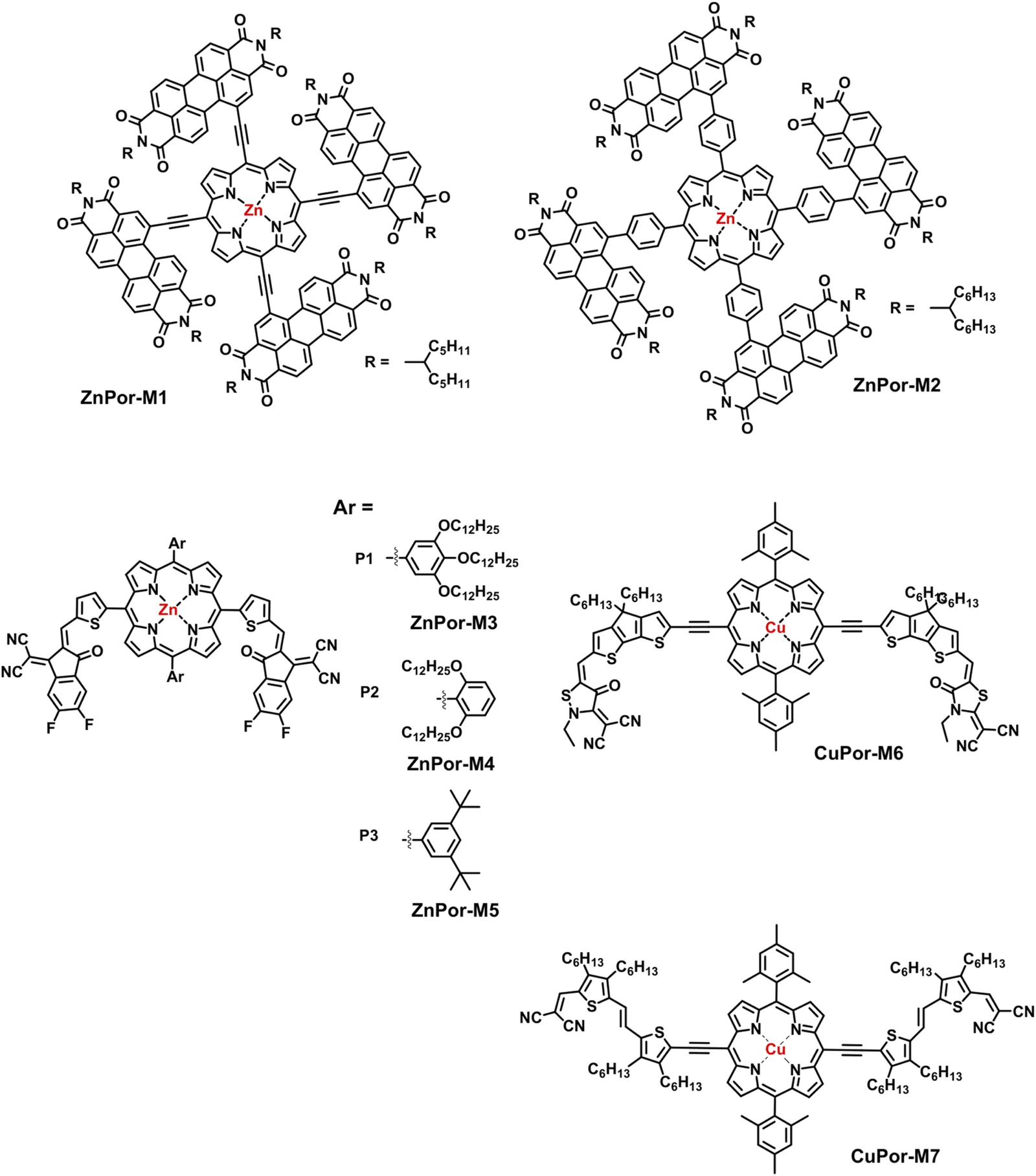
Molecular structures of metalloporphyrin-based NFAs: ZnPor-M1 through ZnPor-M5, AuPor-M6, CuPor-M7, and polymer donors.

The related ZnPor-M2 system, with phenyl bridges replacing ethynyl linkers, demonstrated that linker engineering guided by DFT can significantly impact device performance. Although DFT predicted slightly narrower absorption for ZnPor-M2 due to reduced conjugation, the complementary absorption profile with PTB7-Th resulted in a panchromatic active layer and a higher PCE of 9.40% (*J*_sc_ = 15.35 mA cm^−2^). DFT-optimized geometries revealed that ZnPor-M2 forms highly ordered domains with well-defined π–π stacking, confirmed by AFM and X-ray scattering measurements. The system also exhibited good thermal stability, retaining 51% of initial efficiency after heating at 100 °C for 24 hours.^57^

Tsai *et al.* developed A–D–A porphyrin-based NFAs (ZnPor-M3, ZnPor-M4, ZnPor-M5) from an A_2_B_2_ Zn-porphyrin core with strong electron-withdrawing end groups (2-(5,6-difluoro-3-oxo-2,3-dihydro-1*H*-inden-1-ylidene)malononitrile). DFT calculations predicted low FMO energy levels and a relatively wide band gap of ∼1.5 eV due to the strong electron-accepting terminal moieties, with active layers absorbing broadly from 300 to 900 nm. Crucially, DFT analysis of the meso-substituent effects revealed that alkyl groups strongly influence molecular self-assembly—ZnPor-M4 provides an optimal balance between solubility and controlled π–π stacking, achieving the best PCE of 6.98% (*J*_sc_ = 13.94 mA cm^−2^).^79^

The gold(iii) porphyrin acceptor AuPor-M6, based on the A–π–D–π–A framework with the CPDT unit, represents an important demonstration of how metal exchange tunes acceptor properties. DFT calculations showed that the Au(iii) center, acting as a Lewis acid, stabilizes the LUMO energy level more effectively than Zn(ii), resulting in a deeper LUMO and stronger electron-accepting character. The PTB7-Th: AuPor-M6 device achieved PCE of 9.24% (*J*_sc_ = 17.67 mA cm^−2^), with DFT-guided THF vapor annealing creating optimal phase separation for enhanced charge transport.^58^

DFT calculation revealed almost equal hole and electron mobilities of CuPor-M7 (2.13 × 10^−4^ and 1.98 × 10^−4^ cm^2^ V^−1^ s^−1^) which is the main reason behind its ambipolar character and its ability to perform as either a donor or an acceptor depending on the blending partner. When used as the NFA with PBDB-T, CuPor-M7 achieved a champion binary PCE of 10.84% (*J*_sc_ = 17.42 mA cm^−2^, *V*_oc_ = 1.02 V), while the ternary system PBDB-T: CuPor-M7: Y6 pushed efficiency to 15.25% (*J*_sc_ = 23.47 mA cm^−2^). DFT analysis revealed that the Cu(ii) center provides moderate SOC (∼15 cm^−1^) and nearly balanced reorganization energies for electron and hole transfer (*λ*_e_ ≈ *λ*_h_, Fig. 6b), a rare property that underpins its ambipolar behavior and exceptional device performance.^20^ These parameters are summarized in Table 5.

**Table 5:** Photovoltaic parameters of metalloporphyrin-based OMC NFAs

Acceptor	Donor	*J*_sc_ (mA cm^−2^)	*V*_oc_ (V)	FF (%)	PCE (%)	Ref.
ZnPor-M1	PBDB-T	14.50	0.78	66	7.40	56
ZnPor-M2	PTB7-Th	15.35	0.89	69	9.40	57
ZnPor-M4	PTB7-Th	13.94	0.80	65	6.98	79
AuPor-M6	PTB7-Th	17.67	0.83	63	9.24	58
CuPor-M7 (binary)	PBDB-T	17.42	1.02	61	10.84	80
CuPor-M7: Y6 (ternary)	PBDB-T	23.47	0.89	73	15.25	80
CuPor-M7 (as donor)	Y6	16.38	0.86	56	7.89	80

### 4.5 Subphthalocyanine-based non-fullerene acceptors

Boron subphthalocyanines (SubPcs) are cone-shaped, 14-π-electron aromatic macrocycles with inherent three-dimensional geometry and strong visible absorption. Their non-planar bowl-shaped architecture prevents excessive aggregation in the solid state, making them attractive as both donor and acceptor materials in OSCs. The central boron atom provides a coordination site for axial ligands, enabling fine-tuning of electronic properties and solubility. DFT calculations have been extensively used to understand how axial, peripheral, and metal-center modifications influence SubPc photovoltaic properties.^81–83^

Chen and Peng systematically investigated metal-substituted SubPc systems (Fe, Co, Ni, Cu, Zn centers) paired with C_60_ using DFT/TD-DFT with PCM solvation.^81^ The calculations revealed that stability follows the order Fe > Cu > Ni > Co > Zn, with FeSubPC showing the strongest absorption due to d-electron involvement in the electronic transitions. The experimental ZnSubPC and NiSubPC showed red-shifted absorption in comparison with the boron prototype, and the computed *V*_oc_ (ONi and ZnO) differed greatly from the one of the prototype. The results of this study showed that metal exchange in SubPc is a feasible approach to enhance the electronic performance of NFA, and DFT is a reliable method to predict the relative effect of the different metals.

The effect of axial halogen substitution on the charge transfer (CT) and charge recombination (CR) rate constants of SubPc/C_60_ solar cells was explored using long-range corrected DFT and constrained DFT with Marcus theory for charge transfer rate constants.^82^ Calculations revealed that the identity of the replaced halogen systematically alters the electronic coupling matrix elements and charge transfer (CT) and charge recombination (CR) rate constants for the SubPc-C_60_ binding geometry. Computational studies of the SubPc/C_70_ interface showed that the geometry of the interface significantly influences *V*_oc_ (up to 0.45 V) and exciton lifetimes (70.9 fs–7.81 ps) of a SubPc based device, indicating the importance of the interface geometry for such devices.

Hexachlorinated boron(iii) subphthalocyanine (Cl_6_SubPc) has emerged as one of the most efficient SubPc-based NFAs. DFT/TD-DFT calculations (B3LYP/pcseg-2) revealed that hexachlorination deepens both HOMO and LUMO levels while maintaining strong Q-band absorption, making Cl_6_SubPc an effective electron acceptor in both bilayer and BHJ device architectures^83^ The computed ED_HOMO_–EA_LUMO_ of the Cl_6_SubPc/SubPc pair (1.95 eV) is significantly larger than that of the C_60_/SubPc pair (1.51 eV), predicting a higher *V*_oc_ that has been confirmed experimentally.

Computational design studies have explored SubPc derivatives with various acceptor end-groups. SubPc-based A–D–A architectures with four different end-capped acceptors (X1–X4) were computationally screened at the B3LYP/6-31G(d,p) level, computing absorption spectra, band gaps, charge mobilities, reorganization energies, and transition density matrice.^64^ All designed molecules showed improved characteristics compared to the reference compound. Similarly, seven new diphenylamine-SubPc-based acceptors (N1–N7) were designed using MPW1PW91/6-31G(d,p), showing narrower band gaps, red-shifted absorption, and reduced reorganization energies.^64^ Star-shaped SubPc acceptors with IC end-capped groups (STIC derivatives S1–S6) were also investigated using DFT/TD-DFT, with DOS analysis, FMO visualization, and transition density matrix calculations confirming that peripheral substitution effectively tunes the photovoltaic properties of these three-dimensional twisted acceptor architectures.^65^

### 4.6 BODIPY and azadipyrromethene-based non-fullerene acceptors

Boron-dipyrromethene (BODIPY) and its aza-analogue (aza-BODIPY) represent an important class of boron-coordinated chromophores with strong, narrow absorption bands that can be tuned across the visible-to-NIR spectrum through structural modification. While boron is a metalloid rather than a transition metal, BODIPY complexes share key features with organometallic NFAs including metal-mediated electronic tuning and coordination-driven molecular architecture. DFT calculations have been widely applied to design and optimize BODIPY-based materials for OSC applications.^84–86^

Computational design of BODIPY-based D–A–D molecules for OSCs was reported using DFT/TD-DFT, with three structures (BP-CPDT, BP-DTP, BP-DTS) built around the BODIPY acceptor core flanked by different donor units.^84^ BP-DTS achieved the lowest computed energy gap (0.80 eV), the highest chemical softness (1.25 eV), and the lowest chemical hardness (0.40 eV), indicating the most favorable electron-accepting character. Four D–π–A BODIPY molecular systems were investigated using B3LYP/6-311G(d,p) for geometry optimization and CAM-B3LYP for TD-DFT excitation energies, predicting a maximum PCE of approximately 11.54% when paired with P3HT.^85^ These predictions, while awaiting experimental validation, establish BODIPY-based acceptors as computationally promising candidates for OSC applications.

Aza-BODIPY derivatives have been explored through computational fusion with thiophene units, with DFT/TD-DFT calculations of light-harvesting efficiency, transition density matrices, reorganization energies, and hole/electron transport properties.^86^ Seven new thiophene-fused aza-BODIPY molecules showed superior optoelectronic features compared to experimentally reported compounds, with excellent π-delocalization and stabilizing weak interactions confirmed by computational analysis. Terminal acceptor modification of the aza-BODIPY core (molecules SM1–SM4) was investigated at the B3LYP/6-31G level, showing intense absorption at 710–751 nm with reduced exciton binding energies after electron-withdrawing group incorporation.^87^

Zinc(ii) azadipyrromethene complexes represent true organometallic NFAs combining the strong NIR absorption of the azadipyrromethene chromophore with metal-coordination-driven molecular architecture. Etheridge *et al.* investigated homoleptic Zn(ii) complexes of di(phenylacetylene)azadipyrromethene (Zn(WS3)_2_), computing reorganization energies and molecular geometries by DFT.^88^ The Zn complex adopts a non-planar geometry with low electron transfer reorganization energy, supporting its potential as a fullerene replacement. The absorption maximum at approximately 755 nm with high molar absorptivity (∼100 000 M^−1^ cm^−1^) makes these complexes attractive for NIR photon harvesting. A follow-up study on fluorinated Zn(ii) azadipyrromethene derivatives demonstrated that strategic fluorination increases electron mobility to ∼10^−3^ cm^2^ V^−1^ s^−1^ and reduces bimolecular recombination, with DFT and Raman calculations confirming that fluorination induces a more coplanar backbone geometry that facilitates intermolecular charge transport.^89^

Silicon phthalocyanine ((3BS)_2_-SiPc) has also been demonstrated as a low-cost NFA for flexible large-area OPV devices, with DFT calculations supporting the characterization of its electronic structure and confirming suitable energy level alignment for acceptor applications.^71^ The successful fabrication of 1 cm^2^ flexible devices processed with non-halogenated solvents demonstrates the practical potential of metalloid-coordinated macrocyclic NFAs for scalable OSC technology. These acceptor systems are summarized in Table 6.

**Table 6:** DFT-studied SubPc, BODIPY, and azadipyrromethene-based NFAs

Acceptor type	Representative NFA	DFT method	Key property	PCE (%) or prediction	Ref.
SubPc (B)	Cl_6_SubPc	B3LYP/pcseg-2	Deep LUMO from Cl_6_	Experimental (bilayer)	83
SubPc (metal)	FeSubPC	DFT/TD-DFT/PCM	Strongest absorption (d-electron)	Predicted	81
SubPc (designed)	N1–N7 (DPA-SubPc)	MPW1PW91/6-31G(d,p)	Narrower *E*_g_, red-shifted *λ*	Predicted	64
BODIPY (B)	BP-DTS	DFT/TD-DFT	*E*_g_ = 0.80 eV; low hardness	Predicted	84
BODIPY (B)	D–π–A BODIPY	B3LYP/CAM-B3LYP	Predicted PCE ∼11.54%	∼11.54 (predicted)	85
Zn-azaDPM	Zn(WS3)_2_	DFT	Low *λ*_e_; NIR absorption	Preliminary devices	88
Zn-azaDPM (F)	Fluorinated Zn-aDPM	DFT/Raman	*µ*_e_ ∼10^−3^ cm^2^ V^−1^ s^−1^	Experimental (P3HT)	88
Si-Pc	(3BS)_2_-SiPc	DFT	Low-cost, flexible	Experimental (1 cm^2^)	71

## 5 DFT-derived structure–property relationships and experimental validation

One of the most powerful applications of DFT in the field of OMC-based OSCs is the establishment of quantitative structure–property relationships that correlate computed molecular descriptors with experimental photovoltaic performance. This section presents the key correlations identified from the studies reviewed herein, along with a rigorous assessment of the accuracy of DFT predictions against experimental measurements.

### 5.1 Frontier molecular orbital engineering across metal families

The systematic analysis of DFT-computed FMO energies across all OMC-NFA families reveals clear trends in the influence of metal centers on electronic structure (Fig. 5b). For the families with NFAs, the deepest LUMO level (−3.92 to −4.02 eV) is found in Ferrocene based NFAs (Fc-M1, Fc-M2), which is consistent with the deep LUMO levels of all families considered, and its deepest HOMO level (−5.39 to −5.43 eV), consistent with the strongest electron withdrawing effect that the cyano groups and TCBD groups have. The HOMO values of iridium-based NFAs range from −5.42 eV to −5.45 eV, and the LUMO values range from −3.20 eV to −3.87 eV, suggesting that the Ir complex framework has a moderate electron-accepting character. Platinum NFAs have a broad range of LUMO energies from −3.50 to −3.87 eV as a function of the conjugation length and the strength of the acceptor end group, with the best LUMO of the Pt-M4 covering the optimal range for the acceptors in the window. The characteristic FMO pattern observed in metalloporphyrin NFAs is that the levels of LUMO are in the range of −3.50 to −3.70 eV for Zn-porphyrins (ZnPor-M1, M2, M3, M4, M5), −3.70 eV for Au-porphyrin (AuPor-M6) and −3.72 eV for Cu-porphyrin (CuPor-M7). The correlation between DFT-computed LUMO energy and experimental PCE indicates the optimal LUMO window for producing high PCE (>5%) in binary devices is between −3.5 and −3.9 eV. For the shallower NFA (*e.g.*, Ir-M2, −3.20 eV), the driving force for electron transfer from donor is less, and for the deeper NFA (*e.g.*, Fc-M2, −4.02 eV), the *V*_oc_ falls. This computational guideline provides a rapid screening criterion for new OMC-NFA candidates.

**Fig. 5:**
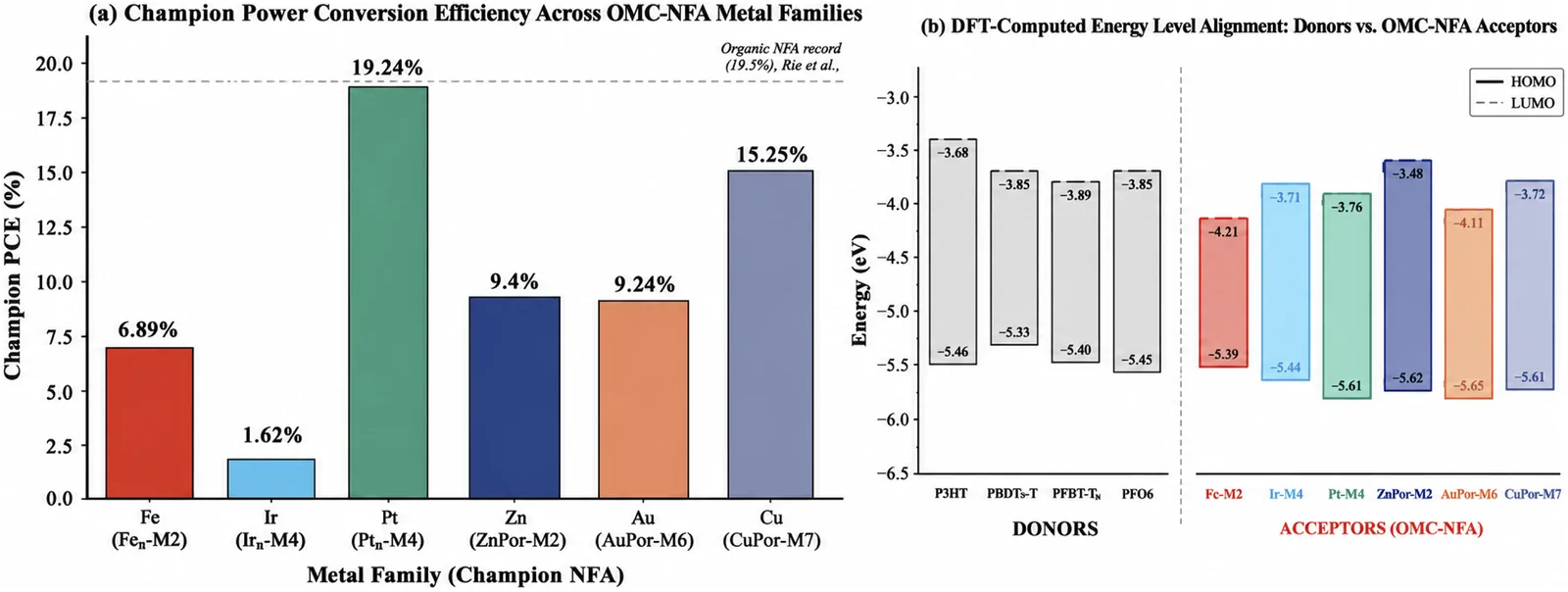
Relationships between structure and properties across metal families of OMC-NFA.

### 5.2 Spin–orbit coupling and charge transfer mechanisms

The heavy metal atoms that are incorporated into the NFA frameworks provide a novel parameter, namely, SOC, which exerts a significant effect on the exciton dynamics and hence the PV efficiency. DFT-computed SOC matrix elements across the OMC-NFA families follow the expected trend with atomic number: Fe (∼5 cm^−1^) < Zn (∼10 cm^−1^) < Cu (∼15 cm^−1^) < Au (∼22 cm^−1^) < Pt (∼40 cm^−1^) < Ir (∼60 cm^−1^) (Fig. 6a).^67^ However, the rate of PCE is non-monotonic with increasing SOC, with the highest binary PCE (10.84%) for CuPor-M7 having moderate SOC (∼15 cm^−1^), and the lowest binary PCE (<2%) for Ir-based NFAs with the highest SOC (∼60 cm^−1^).

**Fig. 6:**
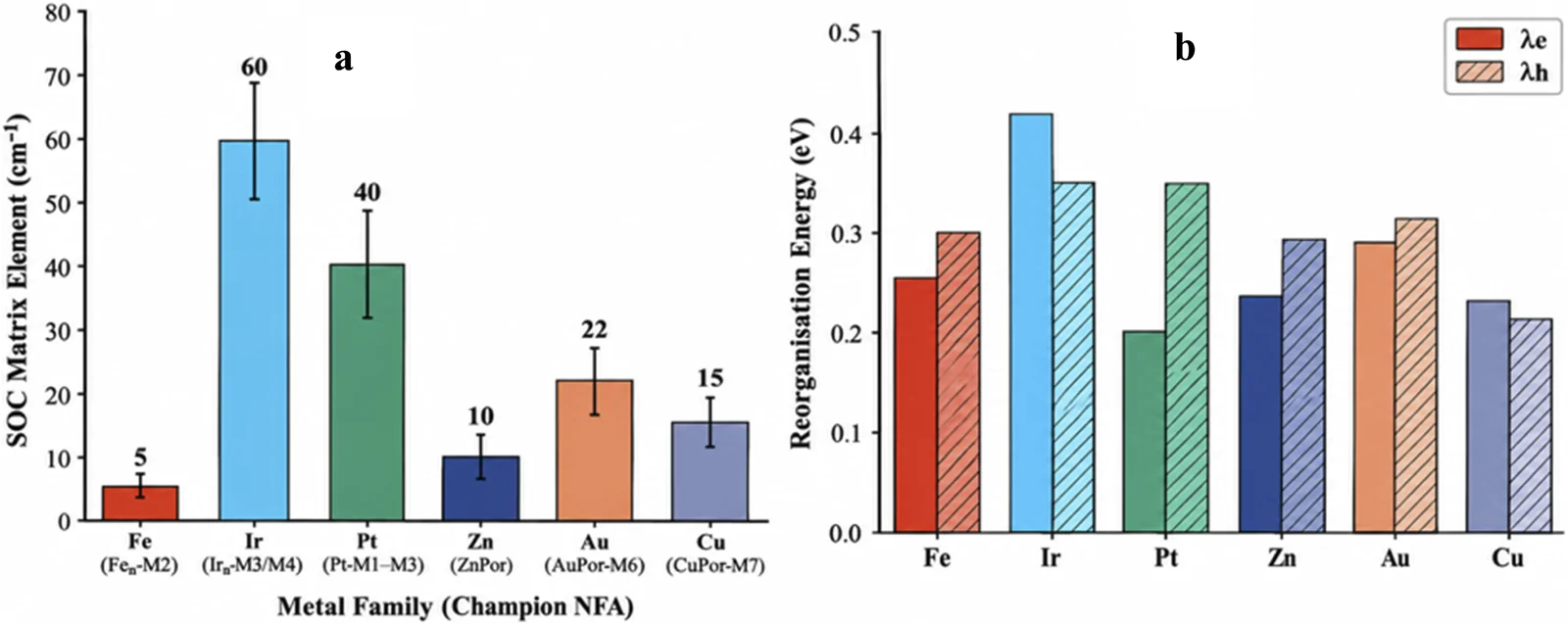
(a) DFT calculation of the spin–orbit coupling (SOC) matrix elements within metals of different OMC families. (b) Marcus–Hush reorganization energies (*λ*_e_ and *λ*_h_) showing the ambipolar nature of CuPor-M7.

The apparent paradox is resolved when the ISC and CRT driving forces for the excess SOC are considered: DFT calculations of the S_1_–T_1_ energy gap and the driving forces of the CRT are consistent with this interpretation, showing that Ir-based NFAs with large SOC exhibit fast ISC, along with fast CRT; Cu-porphyrin NFAs achieve the desired balance of triplet harvesting and recombination suppression. This insight that moderate SOC is optimal for NFA performance was one of the key design principles emerging from computational analysis that would have been hard to deduce from experimental data.^72^

The charge transfer mechanisms in OMC-NFAs are metal-dependent and have been investigated using NTO analysis, transition density matrix calculations, and reduced density gradient analysis (Scheme 1). The predominant ICT character in ferrocene-based NFAs is associated with the Fe d-orbital participation, but with some MLCT character, too. The Ir d-orbitals in the NFAs exhibit both MLCT and LLCT transitions. Platinum NFAs exhibit strong MLCT (metal-to-ligand charge transfer) and SOC-enhanced transitions while metalloporphyrin NFAs show MLCT and π–π* (ligand π–porphyrin π) transitions that are dominated by MLCT.

### 5.3 Comparative analysis: impact of metal centers on photovoltaic performance

The multi-descriptor analysis allows for the overall comparison of all six OMC-NFA metal families where six key parameters |LUMO| energy, electron reorganization energy (*λ*_e_), SOC matrix element, oscillator strength (f), band gap (*E*_g_), and champion PCE are normalized. This analysis shows that each metal family has a unique electronic “fingerprint” and that there are certain compromises that arise in the design of OMC-NFA.

Platinum-based NFAs possess the most balanced profile among all the descriptors with moderate to high value of SOC, |LUMO|, and *λ*_e_, hence their better performance in ternary devices. The Cu-porphyrin NFAs exhibit a characteristic of balanced *λ*_e_/*λ*_h_ and moderate SOC, which leads to their excellent binary PCE. Iridium NFAs have the highest SOC, but have an unfavorable *λ*_e_ and morphological limitations. Ferrocene NFAs have high LUMO depths and low SOC. The fingerprints serve as a quantitative tool to determine the best metal–ligand combination that can be used to get better performance.

There is also an interesting difference between the needs of the binary and ternary device applications indicated by the multi-descriptor analysis. The balance between absorption coverage (oscillator strength), charge transport (reorganization energy) and energy level alignment (|LUMO|) is of particular importance for binary devices, in which the OMC-NFA acts as the main acceptor. The moderate SOC of CuPor-M7 is beneficial in this regime, as it allows for triplet harvesting while minimizing recombination losses, and its ambipolar nature (*λ*_e_ ≈ *λ*_h_) ensures that electron and hole transport are balanced. For ternary devices, however, where the OMC is a morphological and electronic modulator as well as a high-performance organic NFA, the SOC and reorganization energy profiles are more significant than the amount of absorption strength. The phosphorescence features and rigid molecular structure of Pt-M4 minimize the energy disorder and non-radiative energy losses in the ternary blend, whilst the long absorption range of Pt-M4 lets it harvest photons in a complementary fashion without affecting the beneficial morphology of the host PM6: L8-BO system.

One of the most interesting contrasts is between the donor and acceptor applications of the same family of metals. The highest PCEs (more than 16%) have been obtained with iridium complexes as donor materials (PM6Ir1:Y6), while the PCEs of iridium complexes as acceptors (Ir-M3, Ir-M4 with PBDB-T) are lower than 2%. This asymmetry is revealed by DFT analysis to be due to a favorable alignment between the donor and the Ir-complex HOMO for efficient hole transfer in the donor configuration, and the Ir-complex LUMO is too shallow (−3.20 to −3.87 eV *vs.* common donor polymers) for optimal electron acceptance. In contrast, Cu-porphyrin CuPor-M7 has competitive efficiencies as both a donor and an acceptor (7.89% with Y6, 10.84% with PBDB-T), due to its ambipolar character featuring nearly equal HOMO/LUMO offsets compared to both donors and acceptors. These observations, made possible by systematic DFT analysis of the energy level alignment, reveal that optimization strategies for donor and acceptor applications of molecules are very different even within the same metal family. Champion values across all metal families are summarized in Table 7.

**Table 7:** Comparative summary of champion OMC-NFA performance across the metal families with the key DFT insights

Metal family	Champion NFA	Type	Donor	Highest PCE (%)	Key DFT insight
Fe (ferrocene)	Fc-M2	Binary	PD1	6.89	Deep LUMO from TCBD; low SOC
Ir (iridium)	Ir-M4	Binary	PBDB-T	1.62	High SOC but CRT losses
Pt (platinum)	Pt-M4	Ternary	PM6	19.24	Balanced SOC; morphology optimizer
Zn (porphyrin)	ZnPor-M2	Binary	PTB7-Th	9.40	Ordered π–π stacking
Au (porphyrin)	AuPor-M6	Binary	PTB7-Th	9.24	Au(iii) Lewis acid stabilizes LUMO
Cu (porphyrin)	CuPor-M7	Binary	PBDB-T	10.84	Ambipolar; *λ*_e_ ≈ *λ*_h_

## 6 Machine learning and high-throughput DFT screening

This new concept of combining machine learning (ML) with DFT calculations is revolutionizing the discovery process for organic solar cells. As shown in the former sections of the manuscript, previous studies have focused on a particular OMC-NFA system to gain insight into its properties, but recent studies adopt a different strategy, using high throughput DFT screening and ML-driven design to rapidly probe large chemical space for promising candidates. The approach and infrastructure developed in this project for screening approximately 2.3 million candidate molecules for their theoretical PCE, *V*_oc_, and *J*_sc_ on IBM World Community Grid (WCG) distributed computing platform were specifically developed for purely organic materials but have informed approaches to screening organometallic candidates.^73,90,91^

Recently, several studies have used ML models trained using descriptors calculated *via* DFT to predict the photovoltaic properties of organic and organometallic compounds. Meftahi *et al.* demonstrated that ML models leveraging DFT-calculated properties (HOMO, LUMO, *V*_oc_, *J*_sc_) can predict PCE with sufficient accuracy to guide experimental prioritization, establishing quantitative relationships between chemical signatures and OPV properties.^80^ Graph neural networks (GNNs) combined with Light Gradient Boosting Machine (LightGBM) have achieved rapid molecular screening for NFA candidates, while deep learning approaches have enabled *de novo* design of non-fullerene acceptors with predicted performance exceeding current champions.

The DeepAcceptor framework, based on graph representation learning applied to BERT (abcBERT), was pre-trained on over 51 000 NFAs with DFT-computed HOMO/LUMO values and fine-tuned on experimental PCE data.^73^ Three candidates identified through this ML pipeline were subsequently synthesized and characterized, with the best achieving a PCE of 14.61% validating the predictive power of ML-guided NFA design. ML-assisted approaches specifically targeting lower exciton binding energies have used BRICS molecular fragmentation methodology combined with DFT characterization (B3LYP/6-31G(d,p)) to identify 30 NFAs with favorable charge separation properties from a computationally generated library.^90^ A comprehensive review of ML methods in NFA-OSC research published in 2026 highlighted the transformative potential of GNNs, transformers, variational autoencoders (VAEs), generative adversarial networks (GANs), and genetic algorithms for accelerating materials discovery, performance prediction, and molecular design in organic photovoltaics.^91^

Critically for the credibility of the field, several of these campaigns were prospective rather than retrospective: the calculations preceded and directed the experiments instead of rationalizing them after the fact. In the DeepAcceptor study, three acceptor candidates were selected solely by the ML/DFT pipeline and only then synthesized, with the best device delivering 14.61% efficiency.^73^ The Bayesian-optimization study of Ando *et al.* proposed molecules with minimal reorganization energies from only 165 training compounds before any experimental input,^92^ and within the organometallic family the terpolymer donor studies discussed in Section 3.2 progressed from DFT screening of eleven candidate architectures to targeted synthesis of the top-ranked composition. Such computation-led loops remain the exception rather than the rule for OMC-NFAs specifically, and closing this gap between post hoc rationalization and genuine forward design is a central motivation for the metal-specific design rules distilled in Section 5.

Despite these advances, ML-guided screening has not yet specifically targeted organometallic NFAs as a distinct materials class. The unique features of OMC-NFAs including metal-dependent SOC, MLCT/LMCT transitions, and coordination geometry effects require descriptors beyond those used for purely organic systems. The development of metal-aware molecular fingerprints that encode coordination number, ligand field strength, and relativistic effects would enable more accurate ML prediction of OMC-NFA photovoltaic properties. Furthermore, transfer learning approaches that leverage the large existing databases of organic NFA properties to bootstrap predictions for the smaller organometallic dataset could address the data scarcity challenge that currently limits ML application to OMC-based materials.

For organometallic systems specifically, the DFT + ML workflow (Fig. 7) involves: (i) geometry optimization to obtain equilibrium structures; (ii) FMO analysis to compute HOMO/LUMO/*E*_g_; (iii) TD-DFT absorption spectra simulation; (iv) SOC/ISC rate computation; and (v) reorganization energy calculation. These DFT descriptors serve as features for ML models including Random Forest, Gradient Boosting, Transformer-based architectures (abcBERT), and the modified Scharber model, which predict PCE with *R*^2^ > 0.85 and enable virtual screening approximately 100× faster than full DFT calculations.^93,94^ Adaptive Bayesian optimization combined with DFT has also been applied to design molecules with minimal reorganization energies, using only 165 molecules as initial training data to efficiently explore chemical space.^92^

**Fig. 7:**
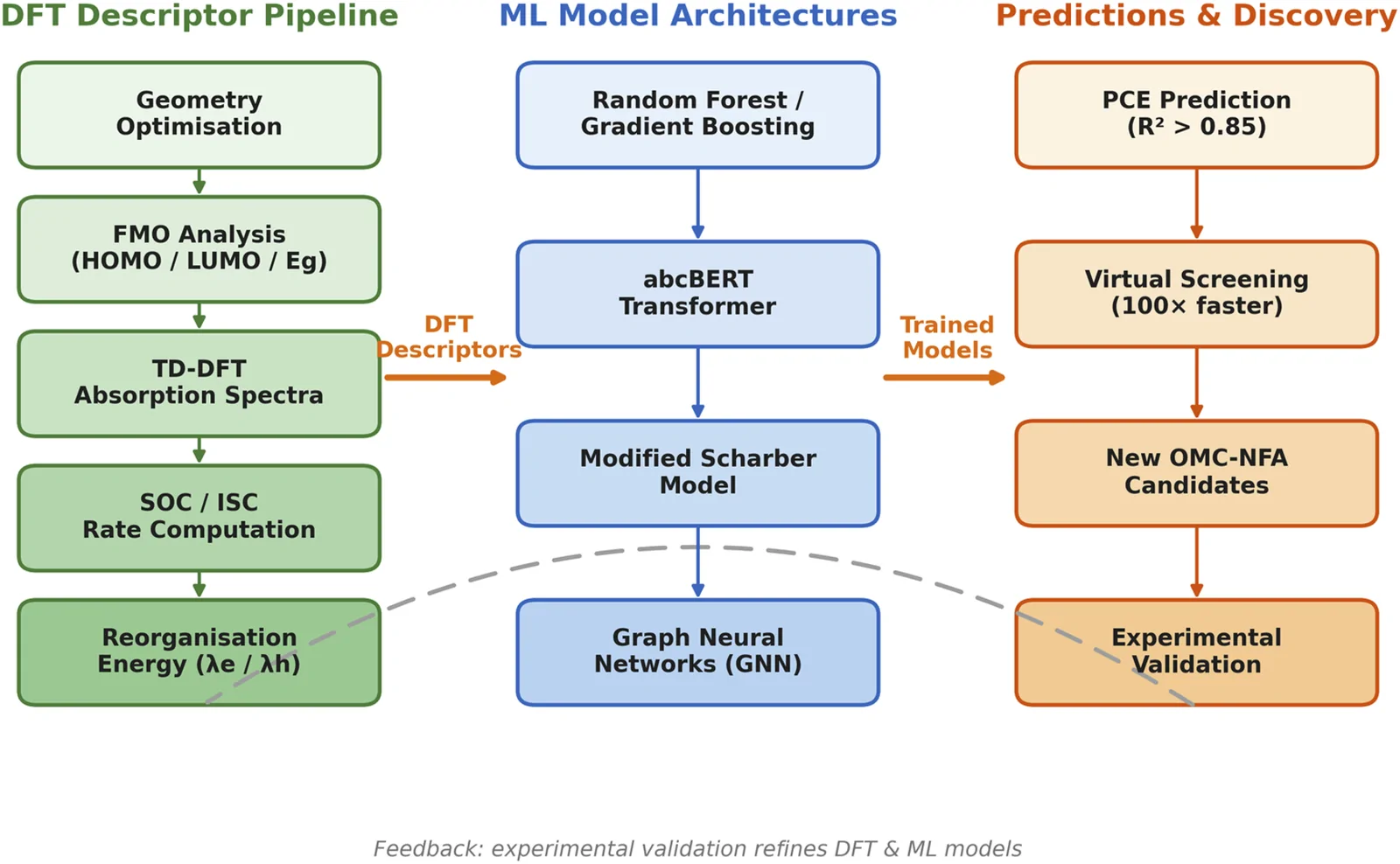
Integrated DFT + AI/ML workflow for OMC-NFA discovery in an accelerated manner, including the DFT descriptor pipeline, ML model architectures, and prediction/discovery results.

A new frontier that is rapidly developing is AI integration into computational and experimental workflow.^95^ For the case of OMC-NFAs, ML-MD, where computational prescreening is followed by synthesis and characterization of the actual materials, will enable a dramatically accelerated exploration of the metal–ligand–acceptor combinatorial space, too large for systematic investigation.

## 7 Challenges and future perspectives

While many advances have been made in DFT-based design of OMC-based OSC materials, there are still many challenges and opportunities. These include computational methodology, materials design, and the mapping of theoretical predictions onto experiments devices.

### 7.1 Computational methodology challenges

The current DFT studies of OMC-NFAs are almost exclusively single molecule gas-phase or implicit-solvation calculations, which describe the electronic properties of individual molecules, but fail to account for the effects of packing into a solid-state material, intermolecular interactions, and morphological properties that play a critical role in determining device properties.^63^ Concrete illustrations of where single-molecule approaches break down are available within the systems reviewed here: gas-phase calculations on isolated CuPc cannot anticipate that the lying-down *versus* standing orientation at the CuPc/C_60_ interface changes the interface dipole and hence the open-circuit voltage,^69^ interface-geometry variations at SubPc/C_70_ heterojunctions shift the predicted *V*_oc_ by up to 0.45 V and alter exciton lifetimes by two orders of magnitude,^82^ and the performance gains from Pt incorporation into NDI polymer acceptors arise from crystallinity and morphology changes that no single-molecule descriptor captures. To provide end-to-end predictions of device performance, there is a need for multi-scale models and computations, where the molecular electronic properties (deep LUMO, strong SOC, broad absorption) of Ir-based NFAs are calculated using DFT, the morphological properties of the film are calculated from molecular dynamics (MD), and the charge transport properties are calculated using kinetic Monte Carlo (kMC) models, while the device performance is calculated using drift-diffusion models and simulations.^95^

However, heavy metal OMCs are still problematic for the treatment of excited states. Scalar relativistic corrections (*via* ECPs) are implemented in standard TD-DFT, thereby allowing the calculation of spin free excitations, but it is not a true treatment of spin forbidden excitations that are relevant for the ISC and triplet harvesting.^96^ Computational research into more efficient ways to calculate SOC and ISC rate for a large organometallic photovoltaic system continues. Other approaches to the problem, for example many-body perturbation theory methods such as the GW approximation and the Bethe–Salpeter equation (BSE), have been developed to give better accuracy for excited states but require much higher computational costs and are not feasible to apply yet to OMC-NFA systems (Fig. 8).

**Fig. 8:**
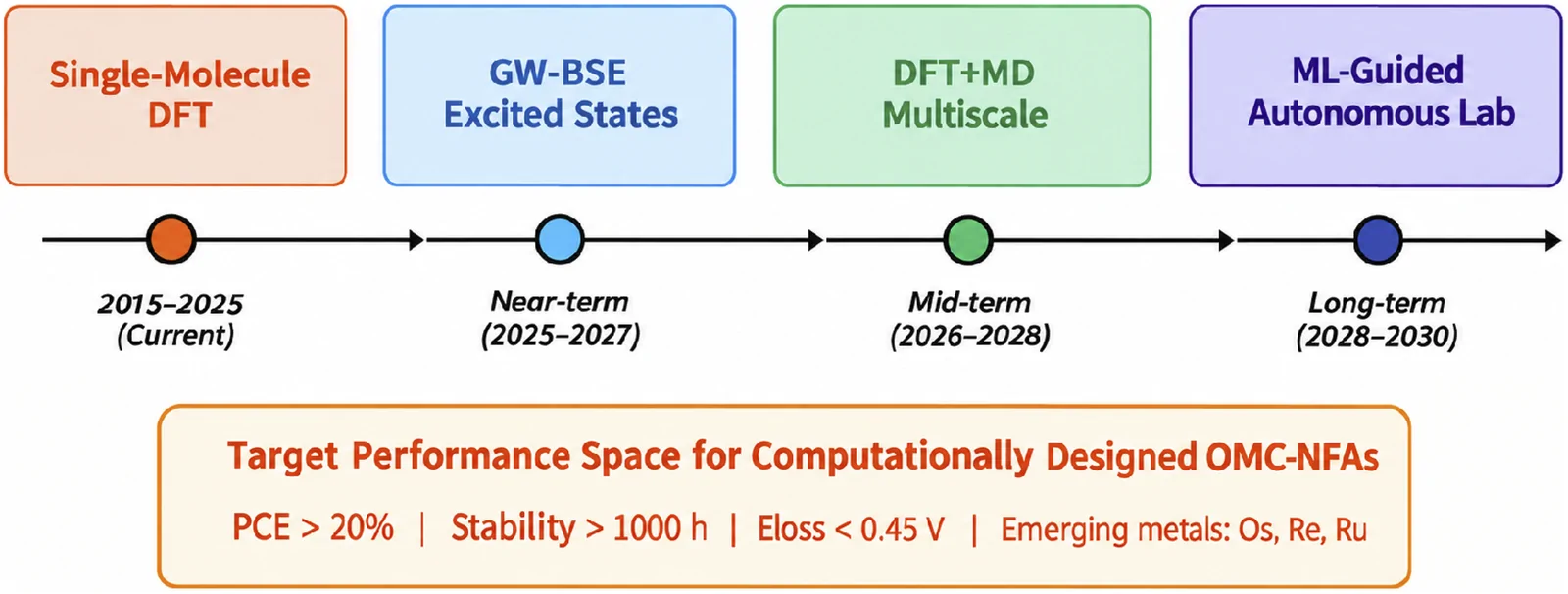
Roadmap for next-generation computational design of OMC-NFAs: single-molecule DFT (2015–2025) → GW-BSE (2025–2027) → DFT + MD multiscale (2026–2028) → ML-guided autonomous laboratories (2028–2030). Target: PCE > 20%, stability > 1000 h, *E*_loss_ < 0.45 V.

One of the major methodological challenges is the ability to obtain an accurate prediction of open-circuit voltage (*V*_oc_) and fill factor (FF) from DFT calculations. Material-specific models based on the DFT-calculated SOC, ISC rates and CT state energies would be required for OMC-NFAs, as the actual voltage losses vary from 0.48 V (CuPor-M7) to 0.70 V (ZnPor-M1), which are significantly different from those of fullerene-based BHJ solar cells.^24^ Beyond-Scharber *V*_oc_ prediction models that include non-radiative recombination *via* metal-mediated pathways are a key methodological frontier. Another practical limitation is the computational expense of studying large organometallic systems.

The development of beyond-Scharber *V*_oc_ prediction models that account for non-radiative recombination through metal-mediated pathways represents an important methodological frontier. Full DFT/TD-DFT calculations on OMC-NFA molecules containing 100–200 atoms with heavy metal ECPs can require days of computation on high-performance clusters, limiting throughput for screening studies. Semi-empirical tight-binding methods (GFN2-xTB) and density-functional tight-binding (DFTB) approaches offer orders-of-magnitude speedups for geometry optimization and can serve as pre-screening filters before full DFT validation. Fragment-based approaches that decompose large OMC-NFA molecules into metal-containing and organic fragments for separate high-level calculations, followed by composite assembly, represent another promising avenue for reducing computational cost while maintaining accuracy for photovoltaic property predictions.

### 7.2 Materials design opportunities

Cost-effective molecular design: the most successful OMC-NFAs involve precious metals (Pt, Ir, Au) that are expensive and scarce. DFT screening of earth-abundant metal alternatives (Cu, Zn, Fe, Mn, Co) with comparable electronic properties could enable cost-effective large-scale production. The success of CuPor-M7 (10.84% PCE with earth-abundant Cu) provides proof-of-concept that precious metals are not essential for high performance.^20^ Computational screening of other first-row transition metals in porphyrin and phthalocyanine frameworks is a promising direction.

Ternary device optimization: the exceptional performance of Pt-M4 as a third component in ternary blends (19.24% PCE) suggests that OMCs may be more effective as morphological and electronic modulators than as primary light-harvesting components.^19^ DFT calculations of interaction energies, miscibility parameters, and domain formation kinetics could guide the rational selection of OMC additives for existing high-performance binary systems. The computational screening of OMC-organic NFA compatibility, including Flory–Huggins interaction parameters from DFT, represents an underexplored but potentially high-impact research direction.

NIR absorption engineering: the inherent ability of OMCs to tune absorption toward the NIR region through MLCT, SOC-enhanced triplet absorption, and Q-band engineering makes them ideal candidates for tandem cells and semitransparent devices. In multi-junctions, OMC-NFAs with an optical band gap below 1.2 eV might be used in conjunction with sub-cells of wide band gap organic or perovskite materials. The roadmap shown in Fig. 8 proposes moving forward from current single-molecule DFT (2015–2025), to GW-BSE excited states (2025–2027), then to DFT + MD multiscale simulations (2026–2028), and finally to ML-guided autonomous laboratories (2028–2030) with targets of PCE > 20%, stability > 1000 h, *E*_loss_ < 0.45 V, and emerging metals Os, Re, and Ru.

Computational studies have been primarily performed on electronic and optical properties, but stability and degradation under sustained illumination is also vital to commercialization of OMC-NFAs. The calculation of metal–ligand bond dissociation energies, pathways for photodegradation and barriers to thermal decomposition, could be of great predictive value for understanding long-term device stability. Preliminary research indicates that certain metalloporphyrin NFAs will maintain 51% efficiency after 24 h at 100 °C though systematic computational studies of the degradation pathways are required.^57^

Dominant metal centers: the studies reviewed herein are dominated by the few metal centers of Fe, Ir, Pt, Zn, Cu, and Au. But the main group and early transition metals show interesting prospects too, as shown by DFT screening. Computational studies of a set of complexes of Ca, Mg and Be with dipyridophenazine acceptors have indicated that the energy gap is reduced by 0.6 eV compared to the metal-free analogues, and that earth-abundant s-block metals could act as effective electronic modulators in the design of NFAs.^97^ Scandium-doped boron nitride (Sc@BN) has been computationally investigated as a non-fullerene acceptor, with an anticipated *V*_oc_ up to 2.3 eV.^98^ Rhenium(i) diimine complexes have been reported to be good organometallic sensitizers for PV cells prepared by vacuum deposition, and DFT calculations of the electronic structure of these complexes have revealed that they have unusual electronic absorption in the range 500–550 nm that is not matched by other parts of the PV cell. A systematic DFT screening of second and third row transition metal complexes (Rh, Pd, Re, W) and lanthanide complexes are a largely unexplored design space that may lead to the discovery of OMC-NFAs with unprecedented sets of SOC, coverage, and stabilities.

Beyond molecular-level design, the donor/acceptor interface is crucial to exciton dissociation, charge transfer and recombination dynamics, and is the subject of Interface Engineering and Charge Dynamics. DFT calculations of model donor: OMC-NFA interfaces including interaction geometries, interface dipoles, charge-transfer state energies, and electronic coupling matrix elements can provide atomistic-level understanding of the factors governing device performance.^69^ For OMC-NFAs, the metal center may introduce additional interfacial states (metal d-orbital hybridization at the heterojunction) that are absent in purely organic systems. Constrained DFT (CDFT) calculations of charge transfer rates at model interfaces, combined with non-adiabatic molecular dynamics simulations of charge separation, could bridge the gap between molecular-level DFT predictions and device-level charge generation efficiency. The development of computationally efficient approaches for studying OMC-NFA/donor polymer interfaces including periodic DFT and QM/MM methods is essential for translating the excellent molecular properties of OMC-NFAs into optimized device architectures.

## 8 Conclusions

This review has provided the first comprehensive assessment of DFT/TD-DFT computational studies on organometallic complexes functioning as both non-fullerene acceptors and donors for organic solar cells. The systematic survey of six donor metal families ferrocene (Fe), iridium (Ir), platinum (Pt), ruthenium (Ru), osmium (Os), and metalloporphyrins (Zn)-together with six NFA metal families (Fe, Ir, Pt, Zn, Au, Cu) several principles in which DFT predictions are borne out by experiment.

First, DFT calculations of the electronic properties are found to be very accurate for OSC materials based on OMC. The computed band gaps correlate well with experimental values, making DFT a good screening tool for the identification of new candidates. Following the correlation analysis between the LUMO and PCE, the optimal LUMO energy window (−3.5 eV to −3.9 eV) gives a fast computational selection of promising OMC-NFA candidates. The recommended computational protocol, B3LYP-D3(BJ)/6-31G(d,p) for geometry optimization and TD-CAM-B3LYP/6-31G(d,p) for absorption spectra in PCM solvent has been tested on all metal families and offers a uniform framework for future comparative studies.

Second, the role of spin–orbit coupling in OMC-NFAs is complicated and metal dependent. High levels of SOC facilitate triplet exciton harvesting, while high levels of SOC leads to charge recombination to triplet (CRT) loss channels. The best SOC regime is found to be moderate values of about 15–40 cm^−1^ for Cu and Pt systems, which is an optimized value to optimize the triplet harvesting against recombination losses. This principle explains the superior performance of Cu-porphyrin (10.84% binary PCE) and Pt-based (19.24% ternary PCE) NFAs compared to Ir-based systems with the highest SOC but lowest device efficiencies.

Third, DFT analysis has established that the most impactful role of OMCs in high-efficiency OSCs may be as third-component additives in ternary blends rather than as primary acceptors in binary devices. The champion efficiency of 19.24% achieved by adding Pt-M4 to the PM6: L8-BO system demonstrates that metal complexes can optimize morphology, suppress non-radiative losses, and enhance charge transport when used as electronic and structural modulators—a function uniquely suited to computational optimization.

Fourth, for donor applications, DFT-guided design of Ir-based complexes has pushed PCEs to 16.71% (PM6Ir1: Y6), while Pd/Pt porphyrin donors achieve remarkable indoor PCEs exceeding 16% under low-light conditions. The computational insights into the configuration-dependent properties of heavy-metal complex donors demonstrating the superiority of three-dimensional tris-chelated geometries over square-planar configurations—provide clear design guidelines for next-generation organometallic donor materials.

Fifth, the emerging classes of OMC-NFAs including subphthalocyanines with tunable axial and peripheral substitutions, BODIPY-based D–A–D architectures with computationally predicted PCEs exceeding 11%, and zinc azadipyrromethene complexes with strong NIR absorption and low reorganization energies significantly expand the chemical space available for OMC-NFA design. DFT screening of metal-substituted SubPcs (Fe, Co, Ni, Cu, Zn centers) has revealed that stability, absorption intensity, and *V*_oc_ vary systematically with the metal identity, providing a computational roadmap for experimental exploration. The silicon phthalocyanine-based NFAs demonstrate that even metalloid-coordinated macrocycles can achieve practical device performance in large-area flexible formats when processed with environmentally benign solvents.

Sixth, the combination of machine learning with DFT calculations is the most promising avenue to speed up OMC-NFA discovery. The DFT + ML workflow developed over the past few years (where the DFT computed descriptors (HOMO, LUMO, *E*_g_, *λ*_e_, *λ*_h_, SOC, *f*) are used as features for ML models such as random forest, gradient boosting, transformer architectures, and graph neural networks) has been found to be able to predict PCE with *R*^2^ > 0.85 and the virtual screening is about 100× faster than full DFT calculation. Nevertheless, there is a significant gap and high-impact opportunity with respect to ML-guided screening that is specifically targeted at organometallic NFAs. The creation of customized DFT databases of OMC-based PV materials, covering a variety of metal centers, ligand structures, and acceptor topologies, would allow for the training of ML models that are tailored to the prediction of the performance of various components of the PV active layer.

In the future, the combination of machine learning and high-throughput screening with DFT is likely to greatly expedite the search for new OSC materials based on OMCs. The combination of multi-scale simulation methods, ranging from single-molecule DFT calculations to modelling of film morphology using molecular dynamics, and autonomous experimental platforms is the most promising route to the development of computationally designed OMC-NFAs that achieve efficiencies above 20% and are stable over time for commercial use. The roadmap outlined in this review envisages a roadmap from the present day of single molecule DFT (2015–2025) to GW-BSE excitation calculations and multiscale DFT + MD calculations (2025–2028) to autonomous laboratories guided by ML (2028–2030) with target performance metrics as PCE > 20%, operational stability > 1000 h, energy loss < 0.45 V, emerging metals with Os, Re, and Ru. Achievement of this roadmap will demand continued investment in methodology development for computation, data curation, and integration of computation with experimental validation; an interdisciplinary effort that has the potential to fundamentally change the way organometallic photovoltaic materials are designed from the empirical trial-and-error process to rational, data-driven discovery.

## Conflicts of interest

The author declares no competing interests.

## Data Availability

Data sharing is not applicable to this article as no new data were created or analyzed in this review.
